# Clinical heterogeneity of feeding and eating disorders: using personality psychopathology to differentiate “simplex” and “complex” phenotypes

**DOI:** 10.1186/s12888-024-06345-3

**Published:** 2024-12-04

**Authors:** Marco Colizzi, Carla Comacchio, Marco Garzitto, Lavinia Bucciarelli, Anna Candolo, Maddalena Cesco, Veronica Croccia, Alessandra Ferreghini, Rosita Martinelli, Alessandra Nicotra, Giulia Sebastianutto, Matteo Balestrieri

**Affiliations:** 1 Unit of Psychiatry and Eating Disorders, Department of Medicine (DMED), University of Udine, Udine, 33100 Italy; 2 Department of Psychosis Studies, Institute of Psychiatry, Psychology and Neuroscience, King’s College London, London, UK

**Keywords:** Anorexia Nervosa, Bulimia Nervosa, Binge Eating Disorder, Mental Health, Symptom clusters, Duration of Untreated Illness, Obsessive–Compulsive Disorder, Anxiety Disorder, Body Mass Index, Personality Psychopathology

## Abstract

**Background:**

To investigate Feeding and Eating Disorders (FED) heterogeneity based on the co-occurrence of FED symptoms and personality psychopathology, on the hypothesis that empirical profiles would not confirm current FED categories but identify unique phenotypes carrying different levels of clinical complexity.

**Methods:**

Latent Profile Analysis profiled FED patients based on the assessment of both FED symptoms, through the Eating Disorders Inventory, third version (EDI-3), and personality characteristics, through the Minnesota Multiphasic Personality Inventory-2. Then, profiles were compared across socio-demographic and clinical characteristics.

**Results:**

Among 109 eligible patients, three FED profiles were identified: (i) FED simplex (low eating symptoms, absence of dysfunctional personality); (ii) FED simplex-severe (high eating symptoms only); and (iii) FED complex-severe (high eating symptoms and dysfunctional personality). Despite an uneven distribution (χ^2^(6) = 15.20, adjusted-*p* = 0.029), FED profiles did not unequivocally confirm clinical diagnoses (e.g., Anorexia Nervosa). A difference in Body Mass Index (BMI) was observed (K(2) = 15.06, adjusted-*p* = 0.001), but lower BMI did not identify the most severe group. Profiles differed in EDI-3 overall scores (e.g., Eating Disorder Risk Composite: K(2) = 43.08, adjusted-*p* < 0.001), Body Uneasiness Test Global Severity Index (GSI: K(2) = 29.33, adjusted-*p* < 0.001), Binge Eating Scale severity (K(2) = 25.49, adjusted-*p* < 0.001), number of psychiatric (K(2) = 8.79, adjusted-*p* = 0.021) and personality diagnoses (K(2) = 11.86, adjusted-*p* = 0.005), and Symptom Checklist-90-Revised GSI (F(2,103) = 37.68, adjusted-*p* < 0.001), with FED complex-severe patients being generally the most severely impaired in terms of FED symptoms, body concerns, depersonalization, and psychiatric comorbidities.

**Conclusions:**

Findings support the hypothesis of distinguishing FED simplex and complex phenotypes, based on the co-occurrence of dysfunctional personality, with implications for FED severity and clinical practice.

**Supplementary Information:**

The online version contains supplementary material available at 10.1186/s12888-024-06345-3.

## Abstract (plain language summary)

Recent years have witnessed relevant changes in diagnostic categories and criteria among Feeding and Eating Disorders (FED), to reduce the frequency with which patients are assigned to a residual category without further specification, which provides little clinical utility. Nevertheless, a substantial within-diagnosis heterogeneity remains, with FED continuing to vary in terms of clinical presentation, treatment response, diagnostic crossover, and course of individual symptoms, leaving their classification partially unsolved. This study used a bottom-up approach to find similar cases and group them to create a given number of maximally different categories. Results support the possibility of distinguishing FED simplex and complex cases, based on whether patients not only present with a severe FED but also with personality problems. As such categories perform differently across several symptomatologic domains, they may orient clinical practice.

**Supplementary Information:**

The online version contains supplementary material available at 10.1186/s12888-024-06345-3.

## Background

Feeding and Eating Disorders (FED) are among the psychiatric disorders that have faced the most relevant modifications in the updated version of the Diagnostic and Statistical Manual of Mental Disorders, Fifth Edition (DSM-5) [1]. The perhaps most relevant aim of such changes was to reduce the frequency with which patients are assigned to the heterogeneous residual category, eating disorder not otherwise specified, which provides little clinical utility [2]. Nevertheless, an elevated within-diagnosis heterogeneity remains, with FED continuing to vary in terms of clinical presentation, treatment response, diagnostic crossover, and course of individual symptoms [3], leaving FED classification still unsatisfactory [4].

To address FED within-diagnosis heterogeneity, with the final goal of improving treatment response, studies have attempted at reclassifying FED based on specific eating patterns [5] and related psychopathology [6, 7] as well as personality comorbidities [3, 8–10]. Intriguingly, such studies have proved a certain utility in predicting FED symptom stability [7] and treatment outcome [11]. On one hand, such evidence has made it clear how complex FED are; on the other, it has revealed how important is to investigate clinical features beyond behaviors strictly related to FED, as they may have different treatment and prognostic implications.

Following the promise of these empirical techniques to better inform FED clinical presentation and treatment outcome, some studies have proposed clusters of personality within FED. In the framework of the Big Five personality model, a study identified three distinct clusters, which were found to be associated with core FED symptoms, with generally greater severity for those patients presenting with undercontrolled/emotionally dysregulated personality [12]. In another study, three personality clusters were also identified, with participants with more dysfunctional personality traits expressing greater FED symptomatology [13]. Other studies used Latent Profile Analysis (LPA) among FED patients. A large sample study proposed a six-profile model of temperaments and characters, capturing both impulsivity and dysfunctional personality traits [14]. In other influential studies, similar empirical methodologies have been applied to AN including temperamental aspects [9] and in Bulimia Nervosa (BN) [10]. Using LPA, a recent study has for the first time integrated both FED symptoms and personality features into a single model to sub-phenotype Anorexia Nervosa (AN) [3]. It has also implemented a dimensional approach, based on the rationale that psychopathology maps on a continuum rather than differentiate groups within a population [15], and may better address FED heterogeneity. As expected, it was found that there are patterns in terms of co-occurrence of personality characteristics and FED psychopathology that help explaining additional within-diagnosis heterogeneity [3]. Interestingly, focusing on aspects of impulsivity and perfectionism, a recent study investigated personality in FED with or without the inclusion of core FED symptoms in the LPA model. Four profiles emerged, with more reliable results when FED symptoms were included into the model [16].

Building up from such previous evidence, the current study aimed at further investigating the notion of FED heterogeneity, without a priori identification of a single diagnostic category (e.g., AN), but across the entire FED population encountered in a specialist service. The entire FED population was modeled based on the co-occurrence of FED symptoms and personality psychopathology, evaluating the relationship between the best-fitting model on one hand, and DSM-5-categories, socio-demographic characteristics, FED severity, and psychiatric comorbidities on the other. We predicted that the best-fitting model would not retrace the DSM-5 categories but rather identify unique phenotypes carrying different levels of clinical complexity, in terms of different levels of psychiatric comorbidity and FED symptoms.

## Methods

### Study design and participants

This study used an observational design and was conducted at the One-stop center for FED of the Unit of Psychiatry of the University Hospital of Udine, Italy. All consecutively assessed patients went through eligibility screening to participate in the study. The inclusion criteria were as follows: (i) diagnosis of a FED in adults according to DSM-5 criteria [17]; and (ii) inclusion in a standardized psychodiagnostic procedure following the agreement of a multidisciplinary team. Out of 193 potentially eligible patients, based on data availability and after excluding a small group of male patients, 111 females with FED were recruited (Supplementary Fig. 1). Assessments were performed by psychiatrists, psychologists, and other health care professionals specialized in FED management, using unstructured and structured interviews as well as psychometric scales. The study was proposed to each consecutive eligible patient by the care team during a routine visit.

### Assessments

#### Eating Disorders Inventory, third version (EDI-3)

The Italian validated version of the EDI-3, was used to assess FED-related psychological symptoms [18, 19]. EDI-3 is probably the most widely used self-report instrument to measure distress associated with FED [20, 21] and has been validated with large samples in multiple languages and countries [22, 23]. It is a Likert-type scale, consisting of 91 items divided into 12 main scales and 6 indices.

Three of the main scales are called specific scales and include Drive for Thinness (DT), Bulimia (B), and Body Dissatisfaction (BD). The remaining nine scales, namely, Low Self-Esteem (LSE), Personal Alienation (PA), Interpersonal Insecurity (II), Interpersonal Alienation (IA), Interoceptive Deficits (ID), Emotional Dysregulation (ED), Perfectionism (P), Asceticism (A), and Maturity Fears (MF) assess psychological aspects especially associated with the development and maintenance of FED. The EDI-3 also allows grouping different scales into six composite indices called: Eating Disorder Risk (EDRC), Global Psychological Maladjustment (GPMC), Ineffectiveness (IC), Interpersonal Problems (IPC), Affective Problems (APC), and Overcontrol (OC). In addition, the EDI-3 has three scales, namely, Negative Impression (NI), Inconsistency (IN), and Infrequency (IF), allowing the analysis of response patterns that suggest a bias in the results. Administered questionnaires did not show severely biased compilations, and none needed to be invalidated.

The three main EDI-3 scales were included in the primary analysis as a measure of FED symptoms.

#### Body Uneasiness Test (BUT)

The Body Uneasiness Test (BUT) was used to assess body image-related distress. It is a self-administered Likert-type questionnaire, initially published in Italian, investigating body shape and/or weight dissatisfaction, avoidance, compulsive control behaviors, feelings of detachment and estrangement toward one’s own body, and worries about specific body parts, shapes, or functions [24, 25]. It presents with 2 parts. BUT-A consists of 34 items divided into 5 subscales, namely, Weight Phobia (WP), Body Image Concerns (BIC), Avoidance (A), Compulsive Self-Monitoring (CSM), and Depersonalization (D), whose scores are then combined in a Global Severity Index (GSI). BUT-B consists of 37 items looking at concerns about specific body parts or functions, whose scores are combined in a global measure, the Positive Symptoms Total (PST), indicating overall dislike of body parts, and a measure of associated distress, Positive Symptom Distress Index (PSDI). Higher scores indicated greater body uneasiness [24, 25].

#### Binge Eating Scale (BES)

The Italian validated version of the Binge Eating Scale (BES) was used to assess binge eating-related distress [26]. It is a 16-item instrument designed to measure the behavioral as well as the emotional and cognitive symptoms associated with binge eating [27]. For each item, respondents are asked to select one of three or four response options, coded zero to two or three, respectively. Possible total scores range from 0 to 46, with higher scores indicating more severe binge eating symptoms. Based on the BES total score, clinical cutoff scores are used to identify none-to-minimal (≤ 17 total score), mild to moderate [18–26], and severe (≥ 27) binge eating problems [27].

#### Structured Clinical Interview for DSM-5—Clinician Version (SCID-5-CV)

The Structured Clinical Interview for DSM-5—Clinician Version (SCID-5-CV) was used to obtain a DSM-5-based standardized diagnosis of psychiatric disorder [28]. The SCID-5-CV is a semi-structured diagnostic interview whose questions allow to investigate each DSM-5 criterion for the diagnoses most commonly encountered in clinical practice, including, but not limited to, depressive and bipolar disorders, schizophrenia spectrum and other psychotic disorders, substance use disorders, anxiety disorders (panic disorder, agoraphobia, social anxiety disorder, generalized anxiety disorder), obsessive–compulsive disorder, posttraumatic stress disorder, attention-deficit/hyperactivity disorder, and adjustment disorder. Seventeen additional DSM-5 disorders can also be screened. By rating each item as either present or absent, a step-by-step diagnosis can be reached.

#### Structured Clinical Interview for DSM-5—Personality Disorder (SCID-5-PD)

The Structured Clinical Interview for DSM-5 Personality Disorders (SCID-5-PD) was used to obtain a DSM-5-based standardized diagnosis of personality disorder [29]. The SCID-5-PD is a semi-structured diagnostic interview whose questions allow to investigate each DSM-5 criterion for the 10 DSM-5 Personality Disorders across Clusters A (Paranoid Personality Disorder, Schizotypal Personality Disorder, Schizoid Personality Disorder), B (Histrionic Personality Disorder, Narcissistic Personality Disorder, Borderline Personality Disorder, Antisocial Personality Disorder), and C (Avoidant Personality Disorder, Dependent Personality Disorder, Obsessive–Compulsive Personality Disorder) as well as Other Specified Personality Disorder. According to the interview, personality disorder diagnoses can be obtained either categorically (present or absent) or dimensionally.

#### Minnesota Multiphasic Personality Inventory-2 (MMPI-2)

The Italian version of the Minnesota Multiphasic Personality Inventory-2 (MMPI-2) was used to assess patients’ personological characteristics [30]. The MMPI-2 is the most widely used psychometric tool for measuring adult psychopathology from a personological perspective in mental health, medical, and employment settings. It is a 567 item, true/false self-report measure, with nine validity scales assessing, among others, for lying, defensiveness, faking good and faking bad. In our sample, no questionnaires had to be invalidated for incorrect completion (i.e., according to the rules in the instrument manual). The test presents with ten main clinical scales assessing dysfunctional personality traits directly associated to mental health problems: Hypochondriasis (Hs), Depression (D), Hysteria (Hy), Psychopathic Deviate (Pd), Masculinity-Femininity (Mf), Paranoia (Pa), Psychasthenia (Pt), Schizophrenia (Sc), Hypomania (Ma), and Social Introversion (Si). The MMPI-2 was developed based on empirical research and not on clinicians’ assumptions of responses potentially indicating specific personality traits [31].

The ten main clinical scales of the MMPI-2 were included in the primary analysis as measure of dysfunctional personality traits.

#### Symptom Checklist-90-Revised (SCL-90-R)

The Italian version of the Symptom Checklist 90-Revised (SCL-90-R) was used to obtain a multifaceted self-report of patients’ psychological distress and psychopathology [32]. It is a widely used Likert-type questionnaire to quickly assess a patient’s type and severity of self-reported symptoms and provide a measure of current psychological status. The SCL-90-R consists of a series of 90 descriptions of symptoms around nine different dimensions: Somatization (SOM), Obsessive–Compulsive (O-C), Interpersonal Sensitivity (I-S; feelings of personal inadequacy and inferiority), Depression (DEP), Anxiety (ANX), Hostility (HOS), Phobic Anxiety (PHOB), Paranoid Ideation (PAR), and Psychoticism (PSY). A global index of distress is also measured, the GSI [33], and the number of reported symptoms and their mean-score are likewise evaluated (respectively, PST and PSDI).

### Data analysis

In univariate analyses, appropriate tests were used for cross tables (i.e., with categories: LPA-derived profiles; FED clinical diagnosis; SCID-5-CV and SCID-5-PD diagnosis) and between-group comparisons (i.e., continuous measures in LPA-derived profiles: age; FED duration; BMI; number of SCID-5-CV and SCID-5-PD diagnosis; scores from EDI-3, BUT, SCL-90-R, and MMPI-2), also taking into consideration any normality or homoscedasticity violation for continuous measures.

For all univariate analyses, effect-sizes were calculated (estimating their magnitude conventionally). We use: φ for Fisher test (small: 0.10 ≤|φ|< 0.30; medium: 0.30 ≤|φ|< 0.50; large: |φ|≥ 0.50); Cramer V for χ^2^-test (based on the minimum number of rows and columns; small: [2] 0.10 ≤|V|< 0.30, [3] 0.07 ≤|V|< 0.21, [> 3] 0.16 ≤|V|< 0.17; medium: [2] 0.30 ≤|V|< 0.50, [3] 0.21 ≤|V|< 0.35, [> 3] 0.17 ≤|V|< 0.29; large: [2] |V|≥ 0.50, [3] |V|≥ 0.35, [> 3] |V|≥ 0.29); Hedges-corrected Cohen d for t-test (small: 0.20 ≤|d|< 0.50; medium: 0.50 ≤|d|< 0.80; large: |d|≥ 0.80); ω^2^ for ANOVA (small: 0.01 ≤|ω^2^|< 0.06; medium: 0.06 ≤|ω^2^|< 0.14; large: |ω^2^|≥ 0.14); Vargha-Delaney A for Mann–Whitney test (small: 0.56 ≤|A|< 0.64; medium: 0.64 ≤|A|< 0.71; large: |A|≥ 0.71); ε^2^ for Kruskal–Wallis test (small: 0.01 ≤|ε^2^|< 0.04; medium: 0.04 ≤|ε^2^|< 0.36; large: |ε^2^|≥ 0.36); r/ρ for Pearson/Spearman correlation (weak: 0.100 ≤|r/ρ|< 0.300; moderate: 0.300 ≤|r/ρ|< 0.700; strong: |r/ρ|≥ 0.700).

Data for LPA were previously standardized in the sample (i.e., in z-scores). Both measures of FED symptoms (i.e., from EDI-3: DT, B, and BD) and measures of dysfunctional personality (i.e., from MMPI-2: Hs, D, Hy, Pd, Mf, Pa, Pt, Sc, Ma, and Si) were included. Multivariate outliers were detected using a proximity matrix with Mahalanobis distance (D^2^) and excluded. LPAs was conducted with mclust-5 software [34]. We preferred to constrain the covariances to zero, testing models with equal variances across profiles (i.e., equal volume and shape, equal orientation) and with free estimated variances (i.e., varying volume and shape, equal orientation). For both highly constrained and more flexible models, solutions with one to 10 profiles were tested. The best solution was selected on the basis on minimization of Bayesian information criterion (BIC) and Akaike information criterion (AIC). The model selection was also guided by the examination of variance explained by Principal Components of the data.

In univariate analyses, missing data were treated with pair-wise selection, otherwise list-wise selection was adopted.

Statistical significance was set at α = 0.050, adopting two-tailed hypotheses. As a total of 102 comparisons were reported for univariate analyses (with 87 independent comparisons, derived from different measures/items), statistical significance was adjusted by Benjamini & Hochberg’s method based on False-Discovery Rate (FDR; reported as adjusted-p). Also, in post-hoc comparisons, statistical significance was corrected with Tukey Honest Significant Differences method (for ANOVA) or with Dunn method (for Kruskal–Wallis test). Given the relatively small sample-size, we indicated the small-size differences as not reliably generalizable, suggesting caution in their interpretation. Analyses were conducted using R-4.3.1 software.

## Results

### Sample socio-demographic and clinical characteristics at a glance

The general description of the sample (*N* = 193) is provided in the Supplementary Table 1. The most frequent FED in the sample was AN (40.5% of the sample), followed by Binge-Eating Disorder (BED; 23.4%), BN (15.3%), Other Specified FED (OSFED; 15.3%), and Unspecified FED (UFED; 5.4%) diagnoses. Details about the samples’ FED diagnoses are reported in the Supplementary Table 2.

In the preliminary data preparation for multivariate analyses (i.e., LPA), two participants were excluded because they were considered multivariate outliers (D^2^: 26 and 77). They were a 28 years-old patient with UFED (Body Mass Index, BMI = 53.7, obesity of third class) and a 19 years-old patient with AN (BMI = 16.8, underweight with moderate thinness), multiple mood and anxiety comorbid diagnoses, and an Avoidant Personality Disorder. Thus, 109 patients were included in the LPA (Supplementary Fig. 1).

### Latent Profile Analysis (LPA)

The LPA was conducted on the EDI-3 risk scales and the MMPI-2 main clinical scales (Table 1), as corrected based on Italian standards. For the highly constrained model, the BIC showed a minimum for the solution with six profiles (BIC = + 3815.92), but this was not confirmed using AIC (+ 3555.80, higher than for the seven-profiles solution). Instead, for the more flexible model, the solution with three profiles was preferable in terms of BIC (+ 3780.72) and AIC (+ 3565.4). Since the 66.2% of the variance in the data was explained by the first three principal components, the only ones with eigenvalues above 1, the model with free estimated variances and three profiles was selected (Supplementary Fig. 2).

**Table 1:** Scales used for the Principal Component Analysis (PCA) and for Latent Profile Analysis (LPA)

	**Mean ± SD [min, Max]**
***EDI-3 Specific scale***	*Standard [%ile]*
DT, *Drive for Thinness*	77.1 ± 20.51 [0, 99]
B, *Bulimia*	69.6 ± 32.45 [0, 99]
BD, *Body Dissatisfaction*	74.9 ± 17.36 [20, 95]
***MMPI-2 scale***	*Standard [z-score]*
Hs, *Hypochondriasis*	+ 1.5 ± 1.24 [−1.4, + 4.3]
D, *Depression*	+ 1.8 ± 1.13 [−1.0, + 3.9]
Hy, *Hysteria*	+ 1.1 ± 1.10 [−2.2, + 4.2]
Pd, *Psychopathic Deviate*	+ 1.5 ± 1.07 [−0.6, + 4.1]
Mf, *Masculinity-Femininity*	+ 0.2 ± 1.20 [−7.9, + 2.7]
Pa, *Paranoia*	+ 1.4 ± 1.26 [−0.76, + 6.1]
Pt, *Psychasthenia*	+ 1.4 ± 1.06 [−1.2, + 3.2]
Sc, *Schizophrenia*	+ 1.3 ± 1.24 [−1.2, + 4.9]
Ma, *Hypomania*	+ 0.2 ± 1.16 [−2.2, + 3.3]
Si, *Social Introversion*	+ 1.1 ± 1.19 [−2.55, + 3.3]

*%ile* Percentile (on the basis of Italian standards), *EDI-3-S* Specific scales of Eating Disorders Inventory, third version, *LPA* Latent Profile Analysis, *MMPI-2* Minnesota Multiphasic Personality Inventory, 2nd version, *PCA* Principal Component Analysis, *z-score* On the basis of Italian standards

**Fig. 1:**
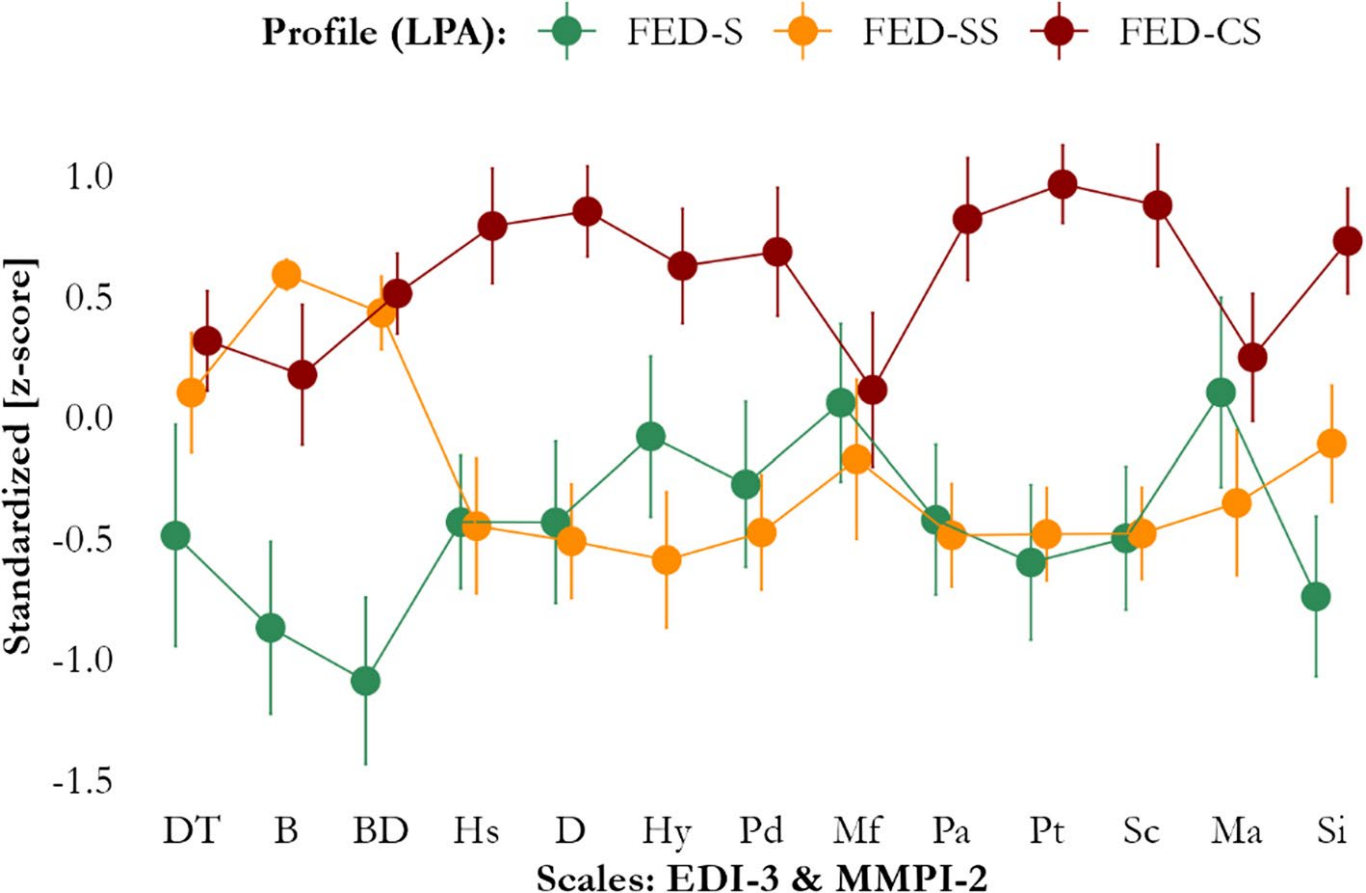
LPA-derived profiles. -CS, “Complex-Severe” FED profile; D, Depression clinical scale (MMPI-2); EDI-3-S, Specific scales of Eating Disorders Inventory, third version; FED, Feeding and Eating Disorders; Hs, Hypochondriasis clinical scale (MMPI-2); Hy, Hysteria clinical scale (MMPI-2); LPA, Latent Profile Analysis; Ma, Hypomania clinical scale (MMPI-2); Mf, Masculinity-Femininity clinical scale (MMPI-2); MMPI-2, Minnesota Multiphasic Personality Inventory, 2nd version; Pa, Paranoia clinical scale (MMPI-2); Pd, Psychopathic Deviate clinical scale (MMPI-2); Pt, Psychasthenia clinical scale (MMPI-2); -S, “Simplex” FED profile; Sc, Schizophrenia clinical scale (MMPI-2); Si, Social Introversion clinical scale (MMPI-2); -SS, “Simplex-Severe” FED profile

Examining LPA profile-associated probabilities, three FED sub-phenotypes were clearly distinguished (Fig. 1). Participants in the first profile (*N* = 33) scored below sample-mean in both the EDI-3 and MMPI-2 scales and were interpreted as having low eating symptoms in the absence of a dysfunctional personality (“FED simplex”). Participants in the second profile (*N* = 37) presented with above sample-mean symptoms in the EDI-3 scales only, with around sample-mean MMPI-2 scores, and were interpreted as having high eating symptoms only (“FED simplex-severe”). Participants in the third profile (*N* = 39), instead, showed high scores at both the EDI-3 and MMPI-2 scales and were interpreted as having both high eating symptoms and a dysfunctional personality (“FED complex-severe”).

### Eating symptom presentation according to the patients’ profile

On arrival at the center, FED simplex participants were younger (mean: 27.7 ± 13.46 years-old) than FED complex-severe (28.9 ± 12.28) and FED simplex-severe ones (35.4 ± 15.87), but not at statistically significant level after FDR adjustment (F(2,106) = 3.19, *p* = 0.045, adjusted-*p* = 0.061). FED simplex-severe participants had a statistically significant longer FED duration than FED simplex (K(2) = 8.04, *p* = 0.018, adjusted-*p* = 0.029; ε^2^ = 0.077 [0.011, 0.212]; 0.015 in post-hoc analysis), but not FED complex-severe ones (*p* = 0.127), without differences between the latter two (*p* = 0.230). Also, considering LPA profiles, a statistically significant difference in BMI was observed (K(2) = 15.06, *p* < 0.001, adjusted-*p* = 0.001; ε^2^ = 0.139 [0.046, 0.301]), with the BMI being higher among FED simplex-severe patients (mean: 26.8 ± 7.82 kg/m^2^) than in FED simplex (21.3 ± 8.56; *p* < 0.001) and FED complex-severe (22.4 ± 7.75; *p* = 0.007) ones. In fact, only 16.2% of FED simplex-severe patients were underweight, with 35.1% of them being obese, while underweight was observed in about half of FED simplex (51.5%) and FED complex-severe (48.7%) patients (χ^2^(2) = 11.84, *p* = 0.003, adjusted-*p* = 0.005; V = 0.330 [0.167, 0.506]).

**Fig. 2:**
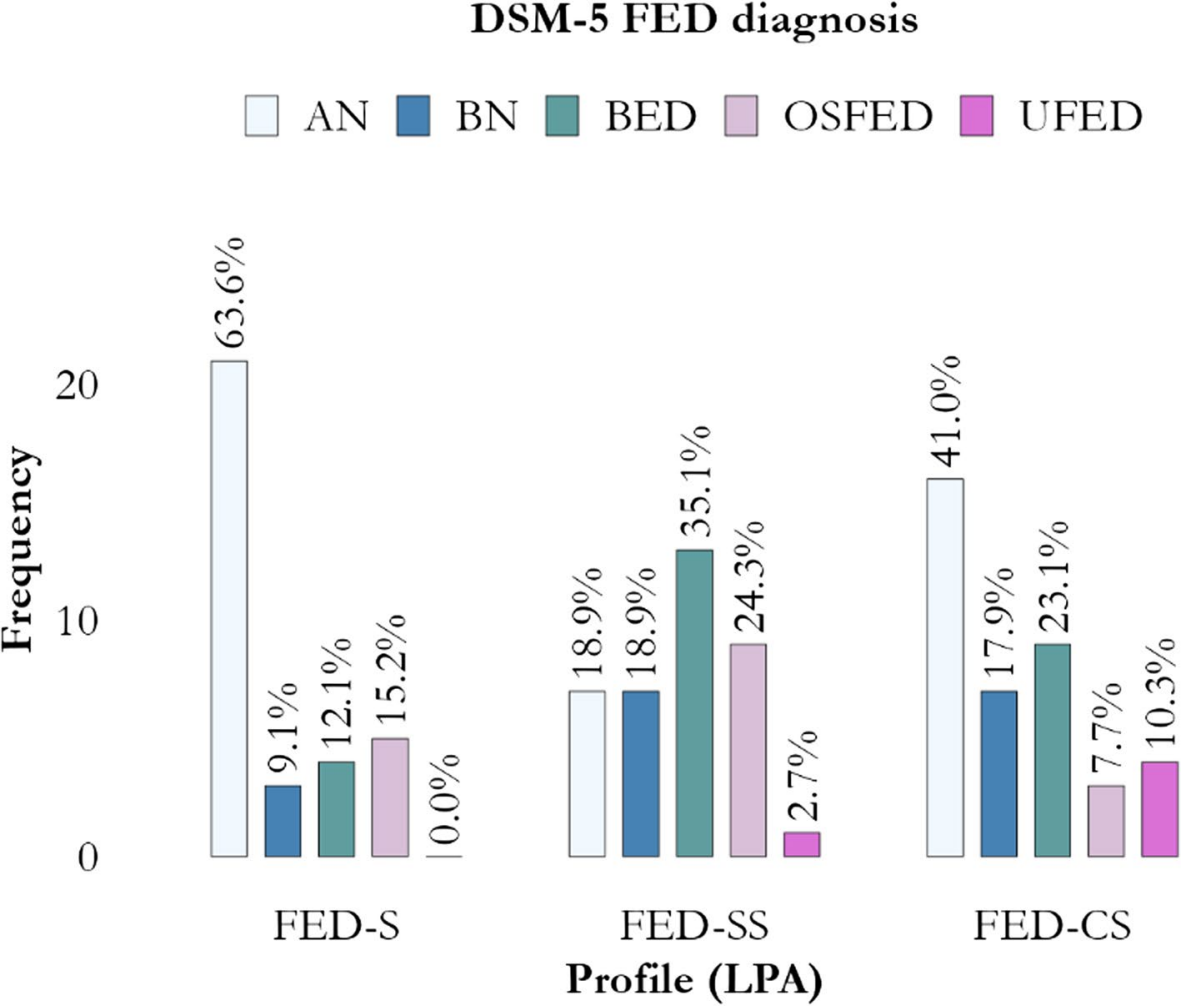
DSM-5 FED diagnosis according to LPA-derived profiles. AN, Anorexia Nervosa; BED, Binge Eating Disorder; BN, Bulimia nervosa; -CS, “Complex-Severe” FED profile; DSM-5, Diagnostic and Statistical Manual of Mental Disorders, Fifth Edition; FED, Feeding and Eating Disorders; LPA, Latent Profile Analysis; OSFED, Other Specified FED; -S, “Simplex” FED profile; -SS, “Simplex-Severe” FED profile; UFED, Unspecified FED

EDI-3 overall scores are reported in the Supplementary Table 3. As expected, except for the MF scale (K(2) = 3.02, *p* = 0.221, adjusted-*p* = 0.278), LPA-derived groups differed in terms of EDI-3 scores (all with *p* ≤ 0.031, adjusted-*p* ≤ 0.043; Table 2). Considering EDRC, a large effect size was observed, with FED simplex-severe and FED complex-severe being similar in their severity (*p* = 0.859 in post-hoc). Interestingly, a moderate effect was also observed for GPMC, with FED simplex and FED simplex-severe being similar in their score (*p* = 0.127 in post-hoc). Complex-severe FED also provided more atypical responses in terms of unpleasantness (NI) of symptoms and more inconsistencies (IN; albeit with a small-size effect). Further, DSM-5-based FED diagnoses were unevenly distributed across the three LPA-derived profiles (χ^2^(6) = 15.20, *p* = 0.019, adjusted-*p* = 0.029), with a moderate effect size (V = 0.264 [0.189, 0.412]; Fig. 2), even though LPA-derived profiles did not unequivocally confirm DSM-5-based diagnoses.

**Table 2:** EDI-3 scores according to LPA-derived profiles

EDI-3	Profile			Comparison
	**FED-S**	**FED-SS**	**FED-CS**	
	n_1_ = 33	n_2_ = 37	n_3_ = 39	Test result; effect-size
***EDI-3-V***				
IN * ^CS>S; CS=SS; SS=S^	−1.0 ± 5.22	+ 1.2 ± 5.38	+ 2.3 ± 4.72	F = 3.8, *p* = 0.026, adjusted-*p* = 0.038; ω^2^ = 0.050 [0.000, 0.143], small effect
IF	0.6 ± 1.03	0.9 ± 1.58	1.3 ± 1.66	K = 6.4, *p* = 0.041, adjusted-*p* = 0.056
NI * ^CS>SS>S^	11.3 ± 9.24	20.2 ± 13.04	28.2 ± 13.46	K = 30.0, *p* < 0.001, adjusted-*p* < 0.001; ε^2^ = 0.278 [0.158, 0.431], moderate effect
Miss	0.3 ± 0.82	0.6 ± 1.54	0.9 ± 1.83	K = 1.38, *p* = 0.501, adjusted-*p* = 0.549
***EDI-3-S***				
DT * ^CS>S; CS=SS; SS=S^	67.3 ± 27.71	79.5 ± 15.84	83.9 ± 13.61	K = 7.5, *p* = 0.024, adjusted-*p* = 0.035; ε^2^ = 0.069 [0.013, 0.212], moderate effect
B * ^CS=SS>S^	41.1 ± 34.27	88.9 ± 6.35	75.3 ± 30.22	K = 30.7, *p* < 0.001, adjusted-*p* < 0.001; ε^2^ = 0.284 [0.144, 0.456], moderate effect
BD * ^CS=SS>S^	56.0 ± 17.73	82.6 ± 8.22	84.0 ± 9.28	K = 45.7, *p* < 0.001, adjusted-*p* < 0.001; ε^2^ = 0.423 [0.256, 0.589], large effect
***EDI-3-C***				
EDRC * ^CS=SS>S^	63.5 ± 19.83	87.5 ± 8.05	87.2 ± 10.08	K = 43.1, *p* < 0.001, adjusted-*p* < 0.001; ε^2^ = 0.399 [0.249, 0.553], large effect
IC * ^CS>SS=S^	64.8 ± 25.77	75.5 ± 15.85	90.8 ± 7.06	K = 37.1, *p* < 0.001, adjusted-*p* < 0.001; ε^2^ = 0.343 [0.212, 0.484], moderate effect
IPC * ^CS>SS=S^	57.0 ± 28.43	70.0 ± 23.82	88.2 ± 11.11	K = 27.7, *p* < 0.001, adjusted-*p* < 0.001; ε^2^ = 0.256 [0.131, 0.400], moderate effect
APC * ^CS>SS=S^	61.0 ± 29.21	69.1 ± 18.90	88.4 ± 10.32	K = 30.7, *p* < 0.001, adjusted-*p* < 0.001; ε^2^ = 0.284 [0.164, 0.440], moderate effect
OC * ^CS=SS>S^	61.6 ± 25.31	76.0 ± 20.54	81.5 ± 18.96	F = 7.9, *p* < 0.001, adjusted-*p* = 0.002; ω^2^ = 0.113 [0.019, 0.227], moderate effect
GPMC * ^CS>SS=S^	51.7 ± 25.19	63.4 ± 18.54	80.3 ± 11.80	K = 31.9, *p* < 0.001, adjusted-*p* < 0.001; ε^2^ = 0.295 [0.166, 0.445], moderate effect
***EDI-3-P***				
LSE * ^CS>SS=S^	62.4 ± 26.10	74.2 ± 18.35	88.7 ± 10.30	K = 33.0, *p* < 0.001, adjusted-*p* < 0.001; ε^2^ = 0.306 [0.169, 0.466], moderate effect
PA * ^CS>SS=S^	63.3 ± 28.12	72.3 ± 19.08	89.7 ± 9.45	K = 30.6, *p* < 0.001, adjusted-*p* < 0.001; ε^2^ = 0.283 [0.149, 0.443], moderate effect
II * ^CS>SS=S^	55.2 ± 29.79	64.5 ± 26.49	84.9 ± 15.13	K = 24.1, *p* < 0.001, adjusted-*p* < 0.001; ε^2^ = 0.223 [0.107, 0.381], moderate effect
IA * ^CS>SS>S^	55.8 ± 28.50	71.0 ± 22.80	84.3 ± 16.13	K = 21.9, *p* < 0.001, adjusted-*p* < 0.001; ε^2^ = 0.203 [0.092, 0.357], moderate effect
ID * ^CS>SS=S^	58.9 ± 29.53	73.0 ± 18.26	89.1 ± 13.94	K = 32.5, *p* < 0.001, adjusted-*p* < 0.001; ε^2^ = 0.300 [0.162, 0.465], moderate effect
ED * ^CS>SS=S^	59.5 ± 31.02	56.5 ± 24.80	78.8 ± 14.64	K = 16.1, *p* < 0.001, adjusted-*p* = 0.001; ε^2^ = 0.149 [0.059, 0.286], moderate effect
P * ^CS=SS>S^	51.8 ± 27.02	67.6 ± 27.69	66.4 ± 32.13	K = 7.0, *p* = 0.031, adjusted-*p* = 0.043; ε^2^ = 0.064 [0.013, 0.180], moderate effect
A * ^CS>SS=S^	63.3 ± 30.46	76.5 ± 17.84	86.4 ± 11.12	K = 12.7, *p* = 0.002, adjusted-*p* = 0.003; ε^2^ = 0.118 [0.037, 0.268], moderate effect
MF	51.3 ± 30.67	57.7 ± 32.38	62.3 ± 32.66	K = 3.0, *p* = 0.221, adjusted-*p* = 0.278

*A* Ascetism (EDI-3-P), *APC* Affective Problems Composite (EDI-3-C), *B* Bulimia (EDI-3-S), *BD* Body Dissatisfaction (EDI-3-S), *-CS* “Complex-Severe” FED profile, *DT* Drive for Thinness (EDI-3-S), *ED* Emotional Dysregulation (EDI-3-P), *EDI-3* Eating Disorders Inventory, third version, *EDI-3-C* Composite scales of EDI-3; EDI-3-P, Psychological scales of EDI-3; EDI-3-S, Specific scales of EDI-3; EDI-3-V, Validity scales of EDI-3, *EDRC* Eating Disorder Risk Composite (EDI-3-C), *ES* Effect Size, *FED* Feeding and Eating Disorders, *GPMC* Global Psychological Maladjustment Composite (EDI-3-C), *IA* Interpersonal Alienation (EDI-3-P), *IC* Ineffectiveness Composite (EDI-3-C), *ID* Interoceptive Deficits (EDI-3-P), *IF* infrequency (EDI-3-V), *II* Interpersonal Insecurity (EDI-3-P), *IN* inconsistency scale (EDI-3-V), *IPC* Interpersonal Problems Composite (EDI-3-C), *LPA* Latent Profile Analysis, *LSE* Low Self-Esteem (EDI-3-P), *MF* Maturity Fears (EDI-3-P), *Miss* Omissions (EDI-3-V), *NI* negative impression (EDI-3-V), *OC* Overcontrol Composite (EDI-3-C), *P* Perfectionism (EDI-3-P), *PA* Personal Alienation (EDI-3-P), *-S* “Simplex” FED profile, *-SS* “Simplex-Severe” FED profile

^*^, Statistically significant after adjustment for false-discovery rate (adjusted-*p* < 0.050; calculated using Benjamini–Hochberg procedure). When appropriate, results of post-hoc tests are shown in superscript: ^CS^, “Complex-Severe” FED profile; ^S^, “Simplex” FED profile; ^SS^, “Simplex-Severe” FED profile

BUT and BES overall scores are reported in the Supplementary Table 4. The three LPA-derived profiles differed at a statistically significant level on all the BUT-A scales (Table 3). Only the effect observed for the CSM scale was of small-size and may not be a particularly prominent characteristic. When considering the GSI, FED complex-severe patients scored higher (2.4 ± 0.83) than both FED simplex-severe (1.9 ± 0.98; *p* = 0.016) and FED simplex (0.9 ± 1.15; *p* < 0.001) patients, with FED simplex-severe patients scoring higher than FED simplex ones (*p* = 0.003). Similar results were observed for the BUT-B scales (Table 3). The PST score was statistically significantly lower in the FED simplex profile (16.1 ± 8.50) than in the FED complex-severe (26.4 ± 8.28; *p* < 0.001) and FED simplex-severe (24.1 ± 9.65; *p* < 0.001) ones, with no statistically significant differences between FED complex-severe and FED simplex-severe profiles (*p* = 0.328).

**Table 3:** BUT and BES according to LPA-derived profiles

	**Profile**			**Comparison**
	**FED-S**	**FED-SS**	**FED-CS**	
	*n*_1_ = 33	*n*_2_ = 37	*n*_3_ = 39	Test result; effect-size
***BUT-A***				
GSI * ^CS>SS>S^	0.9 ± 1.15	1.9 ± 0.98	2.4 ± 0.83	K = 29.3, *p* < 0.001, adjusted-*p* < 0.001; ε^2^ = 0.274 [0.144, 0.446], moderate effect
WP * ^CS=SS>S^	0.6 ± 1.14	1.6 ± 1.05	1.8 ± 0.73	K = 20.9, *p* < 0.001, adjusted-*p* < 0.001; ε^2^ = 0.195 [0.086, 0.343], moderate effect
BIC * ^CS=SS>S^	0.8 ± 1.04	2.0 ± 0.96	2.1 ± 0.79	F = 20.9, *p* < 0.001, adjusted-*p* < 0.001; ω^2^ = 0.269 [0.130, 0.394], large effect
A * ^CS=SS>S^	0.9 ± 1.082	2.2 ± 1.403	2.7 ± 1.46	F = 17.4, *p* < 0.001, adjusted-*p* < 0.001; ω^2^ = 0.233 [0.100, 0.358], moderate effect
CSM * ^CS>S; CS=SS; SS=S^	0.5 ± 1.41	0.8 ± 1.32	1.5 ± 1.32	F = 5.1, *p* = 0.008, adjusted-*p* = 0.014; ω^2^ = 0.070 [0.001, 0.172], small effect
D * ^CS>SS>S^	1.0 ± 1.16	1.8 ± 1.55	2.6 ± 1.23	K = 22.6, *p* < 0.001, adjusted-*p* < 0.001; ε^2^ = 0.211 [0.101, 0.366], moderate effect
***BUT-B***				
PST * ^CS=SS>S^	16.1 ± 8.50	24.1 ± 9.65	26.4 ± 8.28	K = 21.9, *p* < 0.001, adjusted-*p* < 0.001; ε^2^ = 0.202 [0.102, 0.361], moderate effect
PSDI * ^CS=SS>S^	2.4 ± 0.87	2.9 ± 0.77	3.0 ± 0.83	K = 10.7, *p* = 0.005, adjusted-*p* = 0.008; ε^2^ = 0.099 [0.019, 0.242], moderate effect
Mouth * ^CS>SS=S^	1.0 ± 0.79	1.2 ± 0.91	1.9 ± 1.01	K = 15.1, *p* < 0.001, adjusted-*p* = 0.001; ε^2^ = 0.141 [0.047, 0.306], moderate effect
Shape * ^CS>S; CS=SS; SS=S^	0.8 ± 0.99	1.2 ± 1.08	1.5 ± 0.81	K = 14.4, *p* < 0.001, adjusted-*p* = 0.002; ε^2^ = 0.134 [0.038, 0.305], moderate effect
Thighs * ^CS=SS>S^	1.7 ± 1.13	3.4 ± 1.04	3.7 ± 0.93	F = 35.8, *p* < 0.001, adjusted-*p* < 0.001; ω^2^ = 0.392 [0.248, 0.508], large effect
Legs * ^CS=SS>S^	1.1 ± 0.86	2.2 ± 1.29	2.6 ± 0.92	K = 28.5, *p* < 0.001, adjusted-*p* < 0.001; ε^2^ = 0.267 [0.148, 0.410], moderate effect
Harms * ^CS>SS>S^	1.0 ± 0.93	1.9 ± 1.11	2.5 ± 1.12	K = 26.5, *p* < 0.001, adjusted-*p* < 0.001; ε^2^ = 0.247 [0.111, 0.407], moderate effect
* Moustache*	1.1 ± 1.30	1.7 ± 1.63	1.4 ± 1.32	K = 2.2, *p* = 0.326, adjusted-*p* = 0.396
* Skin*	1.4 ± 1.27	1.6 ± 1.45	1.8 ± 1.24	K = 2.6, *p* = 0.278, adjusted-*p* = 342
Blushing * ^CS=SS>S^	1.2 ± 1.00	2.1 ± 1.27	2.4 ± 1.11	K = 20.2, *p* < 0.001, adjusted-*p* < 0.001; ε^2^ = 0.189 [0.078, 0.349], moderate effect
BES * ^CS=SS>S^	10.6 ± 8.89	25.7 ± 10.77	24.1 ± 14.44	K = 25.5, *p* < 0.001, adjusted-*p* < 0.001; ε^2^ = 0.236 [0.137, 0.385], moderate effect

*A* Avoidance (BUT-A), *BES* Binge Eating Scale, *BIC* Body Image Concerns (BUT-A), *BUT* Body Uneasiness Test, *-CS* “Complex-Severe” FED profile, *CSM* Compulsive Self-Monitoring (BUT-A), *D* Depersonalization (BUT-A), *ES* Effect Size, *FED* Feeding and Eating Disorders, *GSI* Global Severity Index (BUT-A), *LPA* Latent Profile Analysis, *PSDI* Positive Symptom Distress Index (BUT-B), *PST* Positive Symptom Total (BUT-B), *-S* “Simplex” FED profile, *-SS* “Simplex-Severe” FED profile, *WP* Weight Phobia (BUT-A)

^*^, Statistically significant after adjustment for false-discovery rate (adjusted-*p* < 0.050; calculated using Benjamini–Hochberg procedure). When appropriate, results of post-hoc tests are shown in superscript: ^CS^, “Complex-Severe” FED profile; ^S^, “Simplex” FED profile; ^SS^, “Simplex-Severe” FED profile

Finally, a statistically significant effect was also observed for the BES (Table 3). Participants with either the FED complex-severe profile (24.1 ± 14.44; *p* < 0.001) and the FED simplex-severe (25.7 ± 10.77; *p* < 0.001) scored higher than those with the FED simplex profile (10.6 ± 8.89). No statistically significant differences were observed between FED complex-severe and FED simplex-severe profiles (*p* = 0.638).

### Comorbid psychological and psychiatric conditions according to the patients’ profile

Out of the sample, 85.1% had at least one diagnosis at the SCID-5-CV, with a maximum of six diagnosis. DSM-5 disorder comorbidities are reported in the Supplementary Table 5, for both SCID-5-CV and SCID-5-PD. LPA-derived profiles differed at a statistically significant level for the number of diagnoses (K(2) = 8.79, *p* = 0.012, adjusted-*p* = 0.021; ε^2^ = 0.103 [0.020, 0.266]), the latter increasing from the FED simplex profile (1.7 ± 1.32 diagnosis) to the FED simplex-severe (2.1 ± 1.52) and FED complex-severe profiles (2.8 ± 1.52), with a statistically significant difference in comparing the FED simplex profile and the FED complex-severe profile in post-hoc analyses (*p* = 0.011). The diagnosis of any anxiety disorder (69.0%) ranged from none to three in the total sample and it also differed significantly according to the LPA-derived profiles (K(2) = 7.23, *p* = 0.027, adjusted-*p* = 0.038; ε^2^ = 0.085 [0.012, 0.248]), with FED simplex patients presenting with less anxiety diagnoses (0.7 ± 0.81) when compared to FED complex-severe patients (1.3 ± 0.94; *p* = 0.030). As reported in Table 4, LPA-derived profiles differed significantly in terms of having any anxiety disorder, Social Anxiety Disorder, and Obsessive–Compulsive Disorder. No other significant differences were observed.

**Table 4:** DSM-5 comorbid diagnosis according to LPA-derived profiles

DSM-5 diagnosis (comorbidity)	Profile			Comparison
	**FED-S**	**FED-SS**	**FED-CS**	
	*n*_1_ = 33	*n*_2_ = 37	*n*_3_ = 39	Test result; effect-size
***SCID-5-CV***				
* Clinical disorder (any)*	75.9%	84.6%	93.6%	χ^2^ = 3.7, *p* = 0.161, adjusted-*p* = 0210
Clinical anxiety disorder (generalized, panic, social) *	48.3%	80.8%	77.4%	χ^2^ = 8.5, *p* = 0.014, adjusted-*p* = 0.024; V = 0.314 [0.121, 0.510], moderate effect
* Psychotic symptoms (no-diagnosis)*	6.9%	-	6.5%	χ^2^ = 1.8, *p* = 0.402, adjusted-*p* = 0.455
* Bipolar disorder*	-	3.9%	6.5%	χ^2^ = 1.9, *p* = 0.393, adjusted-*p* = 0.451
* Depressive disorder*	65.5%	61.5%	80.7%	χ^2^ = 2.8, *p* = 0.244, adjusted-*p* = 0.303
* Generalized anxiety disorder*	37.9	50.0%	45.2%	χ^2^ = 0.8, *p* = 0.661, adjusted-*p* = 0.695
* Panic disorder*	27.6%	38.5%	51.6%	χ^2^ = 3.6, *p* = 0.162, adjusted-*p* = 0.210
Social anxiety disorder *	3.5%	26.9%	32.3%	χ^2^ = 8.3, *p* = 0.016, adjusted-*p* = 0.025; V = 0.311 [0.190, 0.477], moderate effect
* Stress-associated disorder*	10.3%	7.7%	16.1%	χ^2^ = 1.1, *p* = 0.592, adjusted-*p* = 0.635
Obsessive–compulsive disorder *	13.8%	11.5%	38.7%	χ^2^ = 7.8, *p* = 0.020, adjusted-*p* = 0.031; V = 0.302 [0.118, 0.521], moderate effect
* Substance abuse disorder*	-	-	6.5%	χ^2^ = 0.36, *p* = 0.836, adjusted-*p* = 0.861
* Attention-associated disorder*	3.5%	3.9%	6.5%	χ^2^ = 1.2, *p* = 0.559, adjusted-*p* = 0.606
***SCID-5-PD***				
Personality disorder (any) *	34.5%	38.5%	71.0%	χ^2^ = 9.6, *p* = 0.008, adjusted-*p* = 0.014; V = 0.334 [0.167, 0.544], moderate effect
* Personality disorder of cluster A*	10.3%	11.5%	9.7%	χ^2^ = 0.1, *p* = 0.974, adjusted-*p* = 0.984
Personality disorder of cluster B *	10.3%	11.5%	35.5%	χ^2^ = 7.6, *p* = 0.023, adjusted-*p* = 0.038; V = 0.296 [0.095, 0.522], small effect
Personality disorder of cluster C *	20.7%	26.9%	61.3%	χ^2^ = 12.3, *p* = 0.002, adjusted-*p* = 0.002; V = 0.378 [0.190, 0.586], moderate effect
* Paranoid personality disorder*	6.9%	11.5%	6.5%	χ^2^ = 3.4, *p* = 0.487, adjusted-*p* = 0.540
* Schizoid personality disorder*	3.5%	-	3.2%	χ^2^ = 5.8, *p* = 0.219, adjusted-*p* = 0.278
* Schizotypal personality disorder*	-	-	-	χ^2^ < 0.1, *p* = 0.992, adjusted-*p* = 0.992
* Antisocial personality disorder*	3.5%	-	-	χ^2^ = 2.0, *p* = 0.370, adjusted-*p* = 0.429
* Borderline personality disorder*	10.4%	11.5%	35.5%	χ^2^ = 9.1, *p* = 0.058, adjusted-*p* = 0.078
* Histrionic personality disorder*	-	-	3.2%	χ^2^ = 1.8, *p* = 0.771, adjusted-*p* = 0.803
* Narcissistic personality disorder*	-	-	-	χ^2^ = 0.9, *p* = 0.641, adjusted-*p* = 0.681
Avoidant personality disorder *	6.9%	19.2%	51.6%	χ^2^ = 22.9, *p* < 0.001, adjusted-*p* < 0.001; V = 0.365 [0.255, 0.500], large effect
* Dependent personality disorder*	3.5%	3.9%	12.9%	χ^2^ = 4.6, *p* = 0.334, adjusted-*p* = 0.400
* Obsessive–compulsive personality disorder*	13.8%	7.7%	22.6%	χ^2^ = 4.5, *p* = 0.347, adjusted-*p* = 0.411
* Personality disorder NOS*	3.5%	-	-	χ^2^ = 2.0, *p* = 0.370, adjusted-*p* = 0.429

*-CS* “Complex-Severe” FED profile, *DSM-5* Diagnostic and Statistical Manual of Mental Disorders, Fifth Edition, *ES* Effect Size, *FED* Feeding and Eating Disorders, *LPA* Latent Profile Analysis, *NOS* Not Otherwise Specified, *-S* “Simplex” FED profile, *SCID-5-CV* Structured Clinical Interview for DSM-5 Disorders, Clinical Version, *SCID-5-PD* Structured Clinical Interview for DSM-5 Disorders, Personality Disorders, *-SS* “Simplex-Severe” FED profile

^*^, Statistically significant after adjustment for false-discovery rate (adjusted-*p* < 0.050; calculated using Benjamini–Hochberg procedure)

A SCID-5-PD diagnosis of Personality Disorder was attributed to 49.4% of the sample (Supplementary Table 5). The number of Personality Disorder diagnoses ranged from none to four and was higher among FED complex-severe patients (1.4 ± 1.20) than FED simplex (0.5 ± 0.83; *p* = 0.006) or FED simplex-severe (0.5 ± 0.81; *p* = 0.007) ones (K(2) = 11.86, *p* = 0.003, adjusted-*p* = 0.005; ε^2^ = 0.140 [0.036, 0.342]). FED complex-severe patients were more frequently diagnosed with any Personality Disorder (Table 4), Cluster C disorders, Cluster B disorders, and Avoidant Personality Disorder than other profiles. It should be noted that the effect related to the presence of overall cluster B disorders was small in size, and thus it may not be reliable its association with the FED complex-severe profile. No statistically significant differences were observed for Cluster A disorders.

SCL-90-R overall scores are reported in the Supplementary Table 6. Summary scale scores (i.e., GSI, PST, and PSDI; Table 5) as well as all specific scales (i.e., SOM, O-C, I-S, DEP, ANX, HOS, PHOB, PAR, and PSY) differed according to LPA-derived profiles. Specifically, GSI scores were higher among FED complex-severe patients than FED simplex (*p* < 0.001) or FED simplex-severe (*p* < 0.001) ones, with no differences between FED simplex and FED simplex-severe profiles (*p* = 0.972). Also, FED complex-severe patients had higher scores than those with FED simplex or FED simplex-severe profiles in all SCL-90-R scales (all with *p* ≤ 0.043). FED simplex and FED simplex-severe profiles did not show differences in any scale (all with p ≥ 0.459) except for HOS where FED simplex scored higher than FED simplex-severe (*p* = 0.030).

**Table 5:** SCL-90-R according to LPA-derived profiles

SCL-90-R	Profile			Comparison
	**FED-S**	**FED-SS**	**FED-CS**	
	*n*_1_ = 33	*n*_2_ = 37	*n*_3_ = 39	Test result; effect-size
GSI * ^CS>S=SS^	1.1 ± 0.56	1.0 ± 0.46	2.0 ± 0.64	F = 37.7, *p* < 0.001, adjusted-*p* < 0.001; ω^2^ = 0.409 [0.264, 0.524], large effect
PST * ^CS>S=SS^	45.5 ± 21.91	47.1 ± 16.54	69.9 ± 11.89	K = 38.7, *p* < 0.001, adjusted-*p* < 0.001; ε^2^ = 0.358 [0.218, 0.502], moderate effect
PSDI * ^CS>S=SS^	1.8 ± 0.66	1.8 ± 0.59	2.6 ± 0.50	K = 37.6, *p* < 0.001, adjusted-*p* < 0.001; ε^2^ = 0.348 [0.204, 0.497], moderate effect
SOM * ^CS>S=SS^	1.1 ± 0.82	1.0 ± 0.66	1.9 ± 0.93	K = 23.6, *p* < 0.001, adjusted-*p* < 0.001; ε^2^ = 0.224 [0.104, 0.391], moderate effect
O-C * ^CS>S=SS^	1.2 ± 0.68	1.2 ± 0.65	2.3 ± 0.90	K = 30.5, *p* < 0.001, adjusted-*p* < 0.001; ε^2^ = 0.290 [0.153, 0.461], moderate effect
I-S * ^CS>S=SS^	1.2 ± 0.74	1.3 ± 0.70	2.5 ± 0.72	F = 34.4, *p* < 0.001, adjusted-*p* < 0.001; ω^2^ = 0.387 [0.241, 0.504], large effect
DEP * ^CS>S=SS^	1.6 ± 0.81	1.6 ± 0.70	2.7 ± 0.69	K = 37.2, *p* < 0.001, adjusted-*p* < 0.001; ε^2^ = 0.354 [0.211, 0.501], moderate effect
ANX * ^CS>S=SS^	1.0 ± 0.58	0.9 ± 0.57	2.2 ± 0.88	K = 39.5, *p* < 0.001, adjusted-*p* < 0.001; ε^2^ = 0.376 [0.233, 0.533], large effect
HOS * ^CS>S>SS^	0.9 ± 0.72	0.5 ± 0.41	1.3 ± 0.81	K = 20.9, *p* < 0.001, adjusted-*p* < 0.001; ε^2^ = 0.199 [0.089, 0.365], moderate effect
PHOB * ^CS>S=SS^	0.4 ± 0.47	0.5 ± 0.68	1.3 ± 0.95	K = 26.0, *p* < 0.001, adjusted-*p* < 0.001; ε^2^ = 0.248 [0.122, 0.410], moderate effect
PAR * ^CS>S=SS^	1.1 ± 0.81	1.0 ± 0.59	1.9 ± 0.73	F = 19.7, *p* < 0.001, adjusted-*p* < 0.001; ω^2^ = 0.261 [0.122, 0.387], large effect
PSY * ^CS>S=SS^	0.7 ± 0.48	0.6 ± 0.47	1.3 ± 0.58	K = 32.1, *p* < 0.001, adjusted-*p* < 0.001; ε^2^ = 0.306 [0.173, 0.459], moderate effect

*ANX* Anxiety (SCL-90-R), *-CS* “Complex-Severe” FED profile, *DEP* Depression (SCL-90-R), *ES* Effect Size, *FED* Feeding and Eating Disorders, *GSI* Global Severity Index (SCL-90-R), *HOS* Hostility (SCL-90-R), *I-S* Interpersonal Sensitivity (SCL-90-R), *LPA* Latent Profile Analysis, *O-C* Obsessive–Compulsive (SCL-90-R), *PAR* Paranoid Ideation (SCL-90-R), *PHOB* Phobic Anxiety (SCL-90-R), *PSDI* Positive Symptom Distress Index (SCL-90-R), *PST* Positive Symptom Total (SCL-90-R), *PSY* Psychoticism (SCL-90-R), *-S* “Simplex” FED profile, *SCL-90-R* Symptom Checklist, 90-items, Revised version, *SOM* Somatization (SCL-90-R), *-SS* “Simplex-Severe” FED profile

^*^, Statistically significant after adjustment for false-discovery rate (adjusted-*p* < 0.050; calculated using Benjamini–Hochberg procedure). When appropriate, results of post-hoc tests are shown in superscript: ^CS^, “Complex-Severe” FED profile; ^S^, “Simplex” FED profile; ^SS^, “Simplex-Severe” FED profile

MMPI-2 overall scores are reported in the Supplementary Table 7. As expected, most of the MMPI-2 scales differed between LPA-derived profiles (Table 6). Overall, except for the Mf scale (F(2,106) = 0.86, *p* = 0.424, adjusted-*p* = 0.476), FED complex-severe patients scored higher than others on all the scales (*p* ≤ 0.015 in all post-hoc analyses). Instead, FED simplex and simplex-severe profiles performed similarly in post-hoc analyses (all p ≥ 0.248) except for Hy (FED simplex > FED simplex-severe; *p* = 0.041) and Si (FED simplex-severe > FED simplex; *p* = 0.004). Also, FED simplex-severe showed more symptoms (F) and fewer defensive attitudes (K) than the other profiles.

**Table 6:** MMPI-2 according to LPA-derived profiles

MMPI-2	Profile			Comparison
	**FED-S**	**FED-SS**	**FED-CS**	
	*n*_1_ = 33	*n*_2_ = 37	*n*_3_ = 39	Test result; effect-size
***MMPI-2-V***				
L	+ 0.4 ± 1.15	+ 0.2 ± 0.92	−0.1 ± 0.97	K = 4.1, *p* = 0.127, adjusted-*p* = 0.168
F * ^CS>SS=S^	+ 0.5 ± 1.01	+ 0.6 ± 0.81	+ 2.1 ± 1.20	F = 27.0, *p* < 0.001, adjusted-*p* < 0.001; ω^2^ = 0.323 [0.180, 0.445], large effect
K * ^CS<SS=S^	−0.1 ± 1.07	−0.5 ± 0.71	−0.9 ± 0.68	K = 14.0, *p* < 0.001, adjusted-*p* = 0.002; ε^2^ = 0.129 [0.034, 0.296], moderate effect
***MMPI-2-C***				
Hs * ^CS>SS=S^	+ 1.0 ± 1.00	+ 1.0 ± 1.08	+ 2.5 ± 0.95	F = 28.9, *p* < 0.001, adjusted-*p* < 0.001; ω^2^ = 0.338 [0.195, 0.459], large effect
D * ^CS>SS=S^	+ 1.3 ± 1.09	+ 1.2 ± 0.82	+ 2.8 ± 0.66	K = 48.6, *p* < 0.001, adjusted-*p* < 0.001; ε^2^ = 0.450 [0.332, 0.572], large effect
Hy * ^CS>S>SS^	+ 1.1 ± 1.04	+ 0.5 ± 0.93	+ 1.8 ± 0.80	F = 18.9, *p* < 0.001, adjusted-*p* < 0.001; ω^2^ = 0.247 [0.112, 0.372], moderate effect
Pd * ^CS>SS=S^	+ 1.2 ± 1.08	+ 1.0 ± 0.79	+ 2.3 ± 0.91	F = 19.6, *p* < 0.001, adjusted-*p* < 0.001; ω^2^ = 0.255 [0.119, 0.380], moderate effect
Mf	+ 0.3 ± 0.89	+ 0.1 ± 0.95	+ 0.3 ± 0.94	F = 0.9, *p* = 0.424, adjusted-*p* = 0.476
Pa * ^CS>SS=S^	+ 0.9 ± 1.08	+ 0.8 ± 0.78	+ 2.3 ± 0.96	K = 41.2, *p* < 0.001, adjusted-*p* < 0.001; ε^2^ = 0.382 [0.254, 0.525], large effect
Pt * ^CS>SS=S^	+ 0.8 ± 0.98	+ 0.9 ± 0.62	+ 2.4 ± 0.54	K = 62.1, *p* < 0.001, adjusted-*p* < 0.001; ε^2^ = 0.575 [0.470, 0.669], large effect
Sc * ^CS>SS=S^	+ 0.7 ± 1.08	+ 0.7 ± 0.73	+ 2.4 ± 1.00	F = 40.5, *p* < 0.001, adjusted-*p* < 0.001; ω^2^ = 0.420 [0.279, 0.533], large effect
Ma * ^CS>SS; CS=S; SS=S^	+ 0.3 ± 1.32	−0.2 ± 1.07	+ 0.5 ± 0.96	K = 7.8, *p* = 0.020, adjusted-*p* = 0.031; ε^2^ = 0.072 [0.015, 0.201], moderate effect
Si * ^CS>SS>S^	+ 0.3 ± 1.09	+ 1.0 ± 0.84	+ 2.0 ± 0.78	F = 30.3, *p* < 0.001, adjusted-*p* < 0.001; ω^2^ = 0.350 [0.206, 0.470], large effect

***-****CS* “Complex-Severe” FED profile, *D* Depression clinical scale (MMPI-2), *ES* Effect Size, *FED* Feeding and Eating Disorders, *Hs* Hypochondriasis clinical scale (MMPI-2), *Hy* Hysteria clinical scale (MMPI-2), *LPA* Latent Profile Analysis, *Ma* Hypomania clinical scale (MMPI-2), *Mf* Masculinity-Femininity clinical scale (MMPI-2), *MMPI-2* Minnesota Multiphasic Personality Inventory, 2nd version, *MMPI-2-C* Clinical scales of MMPI-2, *MMPI-2-V* Validity scales of MMPI-2, *Pa* Paranoia clinical scale (MMPI-2), *Pd* Psychopathic Deviate clinical scale (MMPI-2), *Pt* Psychasthenia clinical scale (MMPI-2), *-S* “Simplex” FED profile, *Sc* Schizophrenia clinical scale (MMPI-2), *Si* Social Introversion clinical scale (MMPI-2), *-SS* “Simplex-Severe” FED profile

^*^, Statistically significant after adjustment for false-discovery rate (adjusted-*p* < 0.050; calculated using Benjamini–Hochberg procedure). When appropriate, results of post-hoc tests are shown in superscript: ^CS^, “Complex-Severe” FED profile; ^S^, “Simplex” FED profile; ^SS^, “Simplex-Severe” FED profile

## Discussion

This study attempted to profile the entire population of patients with FED cared at a specialist service based on the assessment of both their FED symptoms and personality characteristics. Also, profiles were compared across several socio-demographic and clinical characteristics to further differentiate such groups in terms of clinically meaningful information. In summary, six key findings may be extrapolated, as detailed below.

First, three FED profiles were identified through LPA, that are distinct groups that best represent the patterns in FED-related psychological symptoms, as derived from the EDI-3, and personological characteristics, as derived from the MMPI-2. Even though personality traits have been implicated in the onset, symptomatic expression, and maintenance of FED [35], the current results suggest that not all FED patients present with clinically relevant personality disturbances. In fact, only about one third of patients were found to present with a dysfunctional personality and considered having a complex-severe profile, while the other two groups were characterized by mild or severe FED symptoms only and thus profiled as simplex and simplex-severe patients, respectively. This distinction could encourage personalization of interventions, targeting patients with narrow needs (i.e., mainly oriented to FED symptoms) or with mixed needs (e.g., for whom circumscribed treatment might be ineffective). The proposed profiles were generally consistent with results from previous cluster analysis [12, 13] and LPA [14] studies conducted among the entire FED population. In fact, despite using different methodologies, such previous studies converged on an association between higher dysfunctional personality on one hand, and both core FED symptoms and non-FED psychopathology on the other. However, differently from previous studies, present profiles did not unequivocally identify undercontrolled [12] or impulsive [10, 16] individuals, possibly because the current study was based on standard clinical assessments that did not contemplate the expected theoretical distinction in terms of impulse-control difficulties.

Second, despite an uneven distribution, FED profiles did not unequivocally confirm DSM-5-based diagnoses. Such finding does not mean that the formal FED classification, as articulated in DSM-5, should not be adopted, or did not have clinical utility. However, as widely discussed in the literature [36], such categorical system does not fully acknowledge that many of the FED clinical phenomena exist along a continuum, and, inevitably, there is some degree of arbitrariness in where the boundaries between disorders are drawn. Therefore, a diagnostic approach that integrates true dimensional measures might better account for clinically relevant difficulties, even if sub-threshold or non-diagnostic per se. However, it is worth mentioning that such integration would also involve an inevitable element of arbitrariness. To further complicate things, FED categories are not based on recognition of underlying psychobiological mechanisms, reasons why other nosological approaches, such as the Research Domain Criteria, have been attempted [37]. New nosological approaches are desirable to obtain diagnoses that better capture the causal processes underlying FED.

Third, lower BMI did not necessarily identify the most severe group, with FED complex-severe patients presenting with a BMI intermediate between FED simplex-severe patients (the highest BMI, slightly above normal range) and FED simplex patients (the lowest BMI, but still within normal range). Despite evidence supports the usefulness of BMI as a premorbid metabolic marker of an emerging FED process [38], a recent systematic review and meta-analysis found that change over treatment in BMI does not represent a reliable predictor of outcome [39]. In line with this, results presented here confirm the high levels of heterogeneity across the sample investigated, even after applying the LPA.

Fourth, patients with complex and severe profiles were older at their first access to service, although this difference was not statistically significant, possibly reflecting an effect of the Duration of Untreated Illness (DUI) on FED severity and complexity, worthy of further consideration. In fact, a recent systematic review investigated the average DUI in populations seeking help for FED and its relationship with FED symptom severity and outcome [40]. Interestingly, DUI was found to be DSM-5 diagnosis-dependent but also to influence likelihood of remission and long-term clinical outcome. However, it is worth mentioning that evidence on the association between DUI and outcome comes exclusively from studies on AN [41, 42], urging for further investigations across the wider FED population. Future investigations on larger samples will also be needed to better clarify the possible size of this effect, which in our relatively small sample cannot be reliably generalized.

Fifth, the FED complex-severe profile was associated with more severe FED symptoms, likely signifying that a portion of the increased FED symptomology grounds on a personological substrate. Likewise, body concerns and depersonalization were more severe among those from the FED complex-severe profile. Meta-analytic evidence indicates that personality traits such as elevated negative affectivity, detachment, and conscientiousness may predispose, exacerbate, or maintain dysfunctional eating behaviors [43], resulting in relevant targets to guide clinical practice. There is thus a need for future prospective studies to provide a clearer understanding of the temporal association between personality and FED and whether dysfunctional personality traits may characterize enduring FED and nonresponse [44].

Finally, FED complex-severe patients were more likely to suffer from psychiatric comorbidities, including obsessive–compulsive and anxiety disorders, as well as greater overall psychopathology and personality-related distress. Previous studies have established an increased risk of FED among individuals with other psychiatric disorders and vice versa [45]. Of note, psychiatric comorbidities have been shown to contribute to greater FED symptom severity, maintenance of some FED behaviors, poorer functioning, and worse treatment outcomes [46].

The present findings need to be seen considering their strengths and limitations. On one hand, this report did not focus only on a single disorder, but encompassed several FED commonly encountered in clinical practice. This is probably the main novelty aspect of this study, but the diagnostic heterogeneity of FED would certainly require further investigation on homogeneous samples, especially for the diagnostic categories less studied in the literature (i.e., BED, OSFED, and UFED). Also, to catch the different aspects of a multifaceted phenomenon such as a FED, several investigations were performed at the psychiatric, psychological, and personological level, involving both hetero-evaluation (primarily diagnostic, thus essentially categorical) and self- evaluation (mainly dimensional). On the other, the generalization of the findings may be limited by the fact that FED patients were recruited from a single specialized FED unit and less frequent FED were not represented in terms of DSM-5 diagnoses (e.g., Avoidant-Restrictive Food Intake Disorder). Also, the results cannot be extended to males with FED, as they were excluded in our sample (to avoid a potential distortion in the LPA due to a very small sub-group). Finally, we cannot rule out a role of symptom minimization by patients, especially those with a simplex profile. Denial of disordered eating behaviors is a well acknowledged tendency to conceal symptoms common in all FED [47]. In our study illness denial was not specifically assessed and this may have had an impact on self-evaluations. This topic should certainly be explored in future targeted research.

Taken together, these findings provide evidence to support the hypothesis of distinguishing FED simplex and FED complex phenotypes, based on the co-occurrence of dysfunctional personality. Also, in the context of personality disturbances, patients present with a more severe FED. In conclusion, considering personality traits during the assessment process may help achieving a better understanding of etiological and maintenance factors for FED, ideally guiding a more tailored clinical intervention.

## Supplementary Information

Supplementary Material 1.

## Data Availability

Data available from the corresponding author on request due to restrictions, e.g., privacy or ethical.

## Abbreviations

A: Ascetism
A: Avoidance
AIC: Akaike information criterion
APC: Affective Problems Composite
AN: Anorexia Nervosa
ANX: Anxiety
B: Bulimia
BD: Body Dissatisfaction
BED: Binge Eating Disorder
BES: Binge Eating Scale
BIC: Body Image Concerns
BIC: Bayesian information criterion
BMI: Body Mass Index
BN: Bulimia nervosa
BUT: Body Uneasiness Test
-CS: “Complex-Severe”
CSM: Compulsive Self-Monitoring
D: Depersonalization
D: Depression
DEP: Depression
DSM-5: Diagnostic and Statistical Manual of Mental Disorders, Fifth Edition
DT: Drive for Thinness
DUI: Duration of Untreated Illness
ED: Emotional Dysregulation
EDI-3: Eating Disorders Inventory, third version
EDI-3-C: Composite scales of Eating Disorders Inventory, third version
EDI-3-P: Psychological scales of Eating Disorders Inventory, third version
EDI-3-S: Specific scales of Eating Disorders Inventory, third version
EDI-3-V: Validity scales of Eating Disorders Inventory, third version
EDRC: Eating Disorder Risk Composite
ES: Effect Size
FED: Feeding and Eating Disorders
GPMC: Global Psychological Maladjustment Composite
GSI: Global Severity Index
Hs: Hypochondriasis
Hy: Hysteria
HOS: Hostility
IA: Interpersonal Alienation
IC: Ineffectiveness Composite
ID: Interoceptive Deficits
IF: Infrequency
II: Interpersonal Insecurity
IN: Inconsistency
IPC: Interpersonal Problems Composite
I-S: Interpersonal Sensitivity
LPA: Latent Profile Analysis
LSE: Low Self-Esteem
Ma: Hypomania
MHS: Mental Health Services
MF: Maturity Fears
Mf: Masculinity-Femininity
Miss: Omissions
MMPI-2: Minnesota Multiphasic Personality Inventory, 2nd version
NI: Negative Impression
NOS: Not Otherwise Specified
OC: Overcontrol Composite
O-C: Obsessive-Compulsive
OSFED: Other Specified Feeding and Eating Disorder
P: Perfectionism
PA: Personal Alienation
Pa: Paranoia
PAR: Paranoid Ideation
PCA: Principal Component Analysis
Pd: Psychopathic Deviate
PHOB: Phobic Anxiety
PSDI: Positive Symptom Distress Index
PST: Positive Symptom Total
PSY: Psychoticism
Pt: Psychasthenia
-S: “Simplex”
Sc: Schizophrenia
SCID-5-CV: Structured Clinical Interview for Diagnostic and Statistical Manual of Mental Disorders, Fifth Edition Disorders, Clinical Version
SCID-5-PD: Structured Clinical Interview for Diagnostic and Statistical Manual of Mental Disorders, Fifth Edition Disorders, Personality Disorders
SCL-90-R: Symptom Checklist, 90-items, Revised version
Si: Social Introversion
SOM: Somatization (SCL-90-R)
-SS: “Simplex-Severe”
UFED: Unspecified Feeding and Eating Disorder
WP: Weight Phobia
%ile: Percentile
