# Supplementary material for: Clinical heterogeneity of feeding and eating disorders: using personality psychopathology to differentiate “simplex” and “complex” phenotypes
Source: BMC Psychiatry. 2024 Dec 4;24:888. doi: 10.1186/s12888-024-06345-3 (PMC11616308; doi:10.1186/s12888-024-06345-3)
Supplement: Supplementary file 1 — Supplementary Material 1. [file 12888_2024_6345_MOESM1_ESM.docx]

**Supplementary Figure 1.** Flow-chart of the FED patients included in the LPA and PCA.


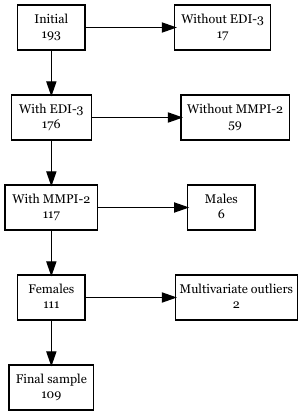


**EDI-3,** Eating Disorders Inventory, third version; **FED,** Feeding and Eating Disorders; **LPA,** Latent Profile Analysis; **MMPI-2,** Minnesota Multiphasic Personality Inventory, 2nd version; **PCA,** Principal Component Analysis.

**Supplementary Figure 2.** Variance explained by PCA (N=109).


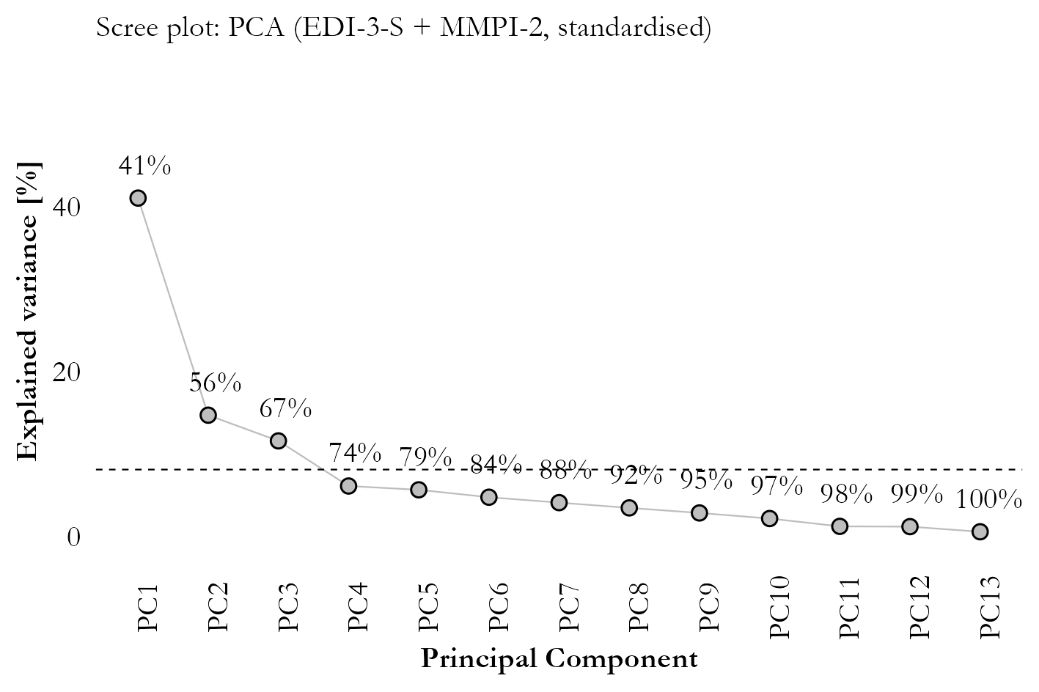


**EDI-3-S,** Specific scales of Eating Disorders Inventory, third version; **MMPI-2,** Minnesota Multiphasic Personality Inventory, 2nd version; **PCA,** Principal Component Analysis.

**Supplementary Table 1.** Socio-demographic and general clinical characteristics (N=111).

| **Measure** | *Missing data* | **N (%)** or **Mean ±SD [min, Max]** |
| --- | --- | --- |
| Age at assessment |  | Age: 30.7 ±14.14 [17, 66] |
| Nationality |  | Italy: 108 (97.3%)  Other: 3 (2.7%) |
| Schooling | 10 | Middle: 34 (33.7%)  High: 48 (47.5%)  Degree: 18 (17.8%)  Post-degree: 1 (1.0%) |
| School failures | 68 | Has any: 14 (32.6%) |
|  |  | Number: 0.5 ±0.80 [0, 3] |
|  |  | Primary school: 0 (0.0%)  Middle school: 6 (14.0%)  High school: 10 (23.3%) |
| Relationship (marital status) | 10 | Single: 71 (70.3%)  Couple: 24 (23.8%)  Separated: 5 (5.0%)  Widowhood: 1 (1.0%) |
| Housing | 5 | Old-family: 68 (64.2%)  New-family: 22 (20.8%)  Alone: 12 (11.3%)  Relatives/Friends: 2 (1.9%)  Other accommodation: 2 (1.9%) |
| Current occupation | 6 | Student: 48 (45.7%)  Employed: 43 (41.0%)  Unemployed: 12 (11.4%)  House-working: 1 (1.0%)  Retired: 1 (1.0%) |
| Source of economical income | 6 | Family: 56 (53.3%)  Autonomous: 45 (42.9%)  Pension: 3 (2.9%)  Other: 1 (1.0%) |
| Medical conditions | 16 | Has any comorbidity: 38 (40.0%) |
|  |  | Has any chronic comorbidity: 33 (34.7%) |
|  |  | Has any partially severe: 14 (14.9%)  Has any severe comorbidity: 10 (10.6%) |
| ***Previous mental health contacts*** |  |  |
| Psychological/Psychiatric support | 12 | Had any: 70 (70.7%) |
| Substance abuse problems | 12 | Had any: 15 (15.2%) |
| Antidepressant | 13 | Prescribed: 27 (27.6%) |
| Mood stabilizer | 13 | Prescribed: 6 (6.1%) |
| Antipsychotics | 13 | Prescribed: 11 (11.2%) |
| Benzodiazepines | 13 | Prescribed: 15 (15.3%) |
| Other drugs | 13 | Prescribed: 19 (19.4%) |
| Previous Services | 14 | Attended any: 55 (56.7%) |
|  |  | Age: 25.1 ±13.13 [12, 54] |
| Previous Child-MHS | 15 | Attended: 12 (12.5%) |
|  |  | Age: 15.7 ±1.38 [14, 18] |
| ***Familial information*** |  |  |
| Psychiatric disorder in family (current) | 19 | No: 40 (43.5%)  One: 30 (32.6%)  More: 22 (23.9%) |
| Psychiatric disorder in close family (current) | 23 | No: 58 (65.9%)  One: 20 (22.7%)  More: 10 (11.4%) |
| Employment-related socio-economic status | 20 | Low: 27 (29.7%)  Medium: 36 (39.6%)  High: 28 (30.8%) |

**MHS,** Mental Health Services.

**Supplementary Table 2.** Eating symptom presentation (N=111).

| **Measure** | *Missing data* | **N (%)** or **Mean ±SD [min, Max]** |
| --- | --- | --- |
| FED DSM-5 diagnosis |  | AN: 45 (40.5%)  BN: 17 (15.3%)  BED: 26 (23.4%)  OSFED: 17 (15.3%)  UFED: 6 (5.4%) |
| Height |  | m: 1.7 ±0.06 [1.5, 1.8] |
| Weight |  | Kg: 65.2 ±24.43 [38.0, 137.5] |
| BMI |  | Kg/m²: 23.8 ±8.73 [13.2, 53.7] |
|  |  | Obesity, class III: 8 (7.2%)  Obesity, class II: 7 (6.3%)  Obesity, class I: 11 (9.9%)  Overweight: 9 (8.1%)  Normal range: 33 (29.7%)  Underweight, mild thinness: 21 (18.9%)  Underweight, moderate thinness: 11 (9.9%)  Underweight, severe thinness: 11 (9.9%) |
| Amenorrhea | 13 | Has: 39 (39.8%) |
| ***General description*** |  |  |
| FED onset | 3 | Age: 17.6 ±7.51 [5, 50] |
| Hospitalization for FED | 8 | Had any: 17 (16.5%) |
|  |  | Age: 23.7 ±10.84 [12, 51] |
|  | 9 | Nose-gastric tube was used: 7 (6.9%) |
| Onset of weight problems | 51 | Age: 20.2 ±12.18 [5, 80] |
| Maximum weight (past) | 3 | Kg: 75.2 ±23.09 [42.0, 151.0] |
|  | 10 | Months ago: 57.2 ±75.38 [0, 408] |
|  | 22 | Duration in months: 23.2 ±46.42 [0, 384] |
| Minimum weight (past) | 4 | Kg: 51.1 ±14.10 [26, 125] |
|  | 15 | Months ago: 82.7 ±121.90 [0, 500] |
|  | 18 | Duration in months: 10.6 ±23.80 [0, 180] |
| Weight more stable, maintained longer over time (past) | 6 | 61.5 ±17.03 [37.0, 135.0] |
|  |  | Age: 22.0 ±8.00 [12, 50] |
| Goal weight | 8 | Reached in the past: 74 (71.8%) |
|  | 37 | Kg: 56.6 ±10.57 [35.0, 80.0] |
|  | 58 | Age: 20.6 ±6.51 [10, 46] |
| Maximum weight loss (past) | 5 | Kg: 15.4 ±10.28 [2, 50] |
|  | 6 | Was voluntary: 90 (85.7%) |
|  | 9 | Kg: 55.4 ±16.57 [25.0, 125.0] |
|  | 12 | Age: 24.6 ±10.95 [5, 59] |
| Estimation of weight without active control | 12 | Kg: 66.3 ±23.74 [38.0, 150.0] |
| Desired weight | 11 | Kg: 54.8 ±10.30 [35.0, 80.0] |
| ***Current interventions*** |  |  |
| Prescribed drugs | 11 | Antidepressant: 53 (53.0%) |
|  |  | Stabilizer: 14 (14.0%) |
|  |  | Antipsychotics: 20 (20.0%) |
|  |  | Benzodiazepines: 19 (19.0%) |
|  |  | Other drugs: 26 (26.0%) |
| Treatment | 9 | Psychiatric treatment: 79 (77.5%) |
|  |  | Psychological treatment: 87 (85.3%) |
|  |  | Group treatment: 11 (10.8%) |
|  |  | Nutritional treatment: 87 (85.3%) |

**AN,** Anorexia Nervosa; **BED,** Binge Eating Disorder; **BMI,** Body mass index; **BN,** Bulimia nervosa; **DSM-5,** Diagnostic and Statistical Manual of Mental Disorders, Fifth Edition; **FED,** Feeding and Eating Disorders; **OSFED,** Other Specified FED; **UFED,** Unspecified FED.

**Supplementary Table 3.** Eating Disorders Inventory, third version (EDI-3).

| **EDI-3** | **N (%)** or **Mean ±SD [min, Max]** |
| --- | --- |
| ***Validity scale*** |  |
| IN  *Inconsistency* | Score [-40, 40]: 0.9 ±5.20 [-10, 12] |
|  | Typical: 35 (32.4%)  Atypical: 73 (67.6%)  Inconsistent: 0 (0.0%) |
| IF  *Infrequency* | Score [0, 10]: 1.0 ±1.48 [0, 8] |
|  | Typical: 98 (88.3%)  Atypical: 13 (11.7%)  Infrequent: 0 (0.0%) |
| NI  *Negative impression* | Score [0, 91]: 20.1 ±13.91 [1, 66] |
|  | Typical: 91 (82.0%)  Atypical: 20 (18.0%)  Negative: 0 (0.0%) |
| Miss  *Omissions* | Number [0, 91]: 0.6 ±1.47 [0, 8] |
|  | Acceptable: 111 (100.0%)  Too many: 0 (0.0%) |
| ***Specific scale*** |  |
| DT  *Drive for Thinness* | Score [0, 28]: 19.9 ±7.77 [0, 28] |
|  | Standard [%ile]: 77.1 ±20.51 [0, 99] |
|  | No clinical relevance (<70): 28 (25.2%)  Clinical relevance (≥70): 33 (29.7%)  High clinical relevance (≥85): 50 (45.1%) |
| B  *Bulimia* | Score [0, 32]: 12.7 ±9.87 [0, 32] |
|  | Standard [%ile]: 69.6 ±32.45 [0, 99] |
|  | No clinical relevance (<70): 36 (32.4%)  Clinical relevance (≥70): 20 (18.0%)  High clinical relevance (≥85): 55 (49.6%) |
| BD  *Body Dissatisfaction* | Score [0, 40]: 25.1 ±7.34 [6, 36] |
|  | Standard [%ile]: 74.9 ±17.36 [20, 95] |
|  | No clinical relevance (<70): 38 (34.2%)  Clinical relevance (≥70): 30 (27.0%)  High clinical relevance (≥85): 43 (38.7%) |
| ***Composite scale*** |  |
| EDRC  *Eating Disorder Risk Composite*  [DT+B+BD] | Score: 57.8 ±18.85 [13, 95] |
|  | Standard [%ile]: 79.9 ±17.07 [27, 99] |
|  | No clinical relevance (<70): 16 (14.4%)  Clinical relevance (≥70): 41 (36.9%)  High clinical relevance (≥85): 54 (48.7%) |
| IC  *Ineffectiveness Composite*  [LSE+PA] | Score: 27.1 ±11.09 [0, 51] |
|  | Standard [%ile]: 77.6 ±20.15 [1, 99] |
|  | No clinical relevance (<70): 28 (25.2%)  Clinical relevance (≥70): 28 (25.2%)  High clinical relevance (≥85): 55 (49.6%) |
| IPC  *Interpersonal Problems Composite*  [II+IA] | Score: 25.4 ±10.83 [3, 50] |
|  | Standard [%ile]: 72.4 ±25.07 [5, 99] |
|  | No clinical relevance (<70): 41 (36.9%)  Clinical relevance (≥70): 19 (17.1%)  High clinical relevance (≥85): 51 (46.0%) |
| APC  *Affective Problems Composite*  [ID+ED] | Score: 25.8 ±13.11 [0, 56] |
|  | Standard [%ile]: 73.4 ±23.19 [1, 99] |
|  | No clinical relevance (<70): 41 (36.9%)  Clinical relevance (≥70): 22 (19.8%)  High clinical relevance (≥85): 48 (43.2%) |
| OC  *Overcontrol Composite*  [P+A] | Score: 21.0 ±9.37 [3, 38] |
|  | Standard [%ile]: 73.0 ±23.58 [7, 98] |
|  | No clinical relevance (<70): 42 (37.8%)  Clinical relevance (≥70): 19 (17.1%)  High clinical relevance (≥85): 50 (45.1%) |
| GPMC  *Global Psychological Maladjustment Composite*  [IC+IPC+APC+OC+MF] | Score: 87.3 ±32.48 [7, 158] |
|  | Standard [%ile]: 65.6 ±22.16 [1, 96] |
|  | No clinical relevance (<70): 54 (48.7%)  Clinical relevance (≥70): 31 (27.9%)  High clinical relevance (≥85): 26 (23.4%) |
| ***Psychological scale*** |  |
| LSE  *Low Self-Esteem* | Score [0, 24]: 13.8 ±5.91 [0, 24] |
|  | Standard [%ile]: 75.6 ±21.49 [0, 99] |
|  | No clinical relevance (<70): 29 (26.1%)  Clinical relevance (≥70): 39 (35.1%)  High clinical relevance (≥85): 43 (38.7%) |
| PA  *Personal Alienation* | Score [0, 28]: 13.3 ±6.08 [0, 27] |
|  | Standard [%ile]: 75.7 ±22.35 [0, 99] |
|  | No clinical relevance (<70): 30 (27.0%)  Clinical relevance (≥70): 33 (29.7%)  High clinical relevance (≥85): 48 (43.2%) |
| II  *Interpersonal Insecurity* | Score [0, 28]: 13.0 ±6.65 [0, 28] |
|  | Standard [%ile]: 69.0 ±26.92 [0, 99] |
|  | No clinical relevance (<70): 47 (42.3%)  Clinical relevance (≥70): 24 (21.6%)  High clinical relevance (≥85): 40 (36.0%) |
| IA  *Interpersonal Alienation* | Score [0, 28]: 12.4 ±5.27 [1, 23] |
|  | Standard [%ile]: 71.0 ±25.13 [3, 99] |
|  | No clinical relevance (<70): 45 (40.5%)  Clinical relevance (≥70): 19 (17.1%)  High clinical relevance (≥85): 47 (42.3%) |
| ID  *Interoceptive Deficits* | Score [0, 36]: 16.7 ±9.10 [0, 36] |
|  | Standard [%ile]: 74.4 ±24.08 [0, 99] |
|  | No clinical relevance (<70): 36 (32.4%)  Clinical relevance (≥70): 26 (23.4%)  High clinical relevance (≥85): 49 (44.1%) |
| ED  *Emotional Dysregulation* | Score [0, 32]: 9.0 ±5.40 [0, 22] |
|  | Standard [%ile]: 65.1 ±25.78 [0, 98] |
|  | No clinical relevance (<70): 55 (49.6%)  Clinical relevance (≥70): 27 (24.3%)  High clinical relevance (≥85): 29 (26.1%) |
| P  *Perfectionism* | Score [0, 24]: 9.6 ±5.47 [0, 20] |
|  | Standard [%ile]: 62.0 ±29.88 [0, 98] |
|  | No clinical relevance (<70): 52 (46.9%)  Clinical relevance (≥70): 25 (22.5%)  High clinical relevance (≥85): 34 (30.6%) |
| A  *Ascetism* | Score [0, 28]: 11.5 ±5.61 [0, 26] |
|  | Standard [%ile]: 75.4 ±23.34 [0, 99] |
|  | No clinical relevance (<70): 35 (31.5%)  Clinical relevance (≥70): 22 (19.8%)  High clinical relevance (≥85): 54 (48.7%) |
| MF  *Maturity Fears* | Score [0, 32]: 13.5 ±7.80 [0, 32] |
|  | Standard [%ile]: 57.7 ±31.87 [0, 99] |
|  | No clinical relevance (<70): 63 (56.8%)  Clinical relevance (≥70): 14 (12.6%)  High clinical relevance (≥85): 34 (30.6%) |

**%ile,** Percentile (on the basis of Italian standards); **EDI-3,** Eating Disorders Inventory, third version.

**Supplementary Table 4.** Body Uneasiness Test (BUT) and Binge Eating Scale (BES).

|  | **N (%)** or **Mean ±SD [min, Max]** |
| --- | --- |
| ***BUT, Part A*** |  |
| GSI  *Global Severity Index* | Score [0, 5]: 2.7 ±1.02 [0.4, 4.5] |
|  | Standard [z-score]: 1.8 ±1.17 [-1.2, +4.3] |
|  | Norm (<1.5): 38 (34.6%)  Subclinical (≥1.5): 22 (20.0%)  Clinical (≥2.0): 50 (45.5%) |
| WP  *Weight Phobia* | Score [0, 5]: 3.3 ±1.21 [0.4, 5.0] |
|  | Standard [z-score]: 1.3 ±1.11 [-1.3, +4.0] |
|  | Norm (<1.5): 55 (50.0%)  Subclinical (≥1.5): 22 (20.0%)  Clinical (≥2.0): 33 (30.0%) |
| BIC  *Body Image Concerns* | Score [0, 5]: 3.2 ±1.13 [0.2, 5.0] |
|  | Standard [z-score]: 1.6 ±1.09 [-1.2, +4.3] |
|  | Norm (<1.5): 43 (39.1%)  Subclinical (≥1.5): 20 (18.2%)  Clinical (≥2.0): 47 (42.7%) |
| A  *Avoidance* | Score [0, 5]: 2.1 ±1.16 [0.0, 4.7] |
|  | Standard [z-score]: 2.0 ±1.53 [-0.7, +6.5] |
|  | Norm (<1.5): 49 (44.6%)  Subclinical (≥1.5): 7 (6.4%)  Clinical (≥2.0): 54 (49.1%) |
| CSM  *Compulsive Self-Monitoring* | Score [0, 5]: 2.2 ±1.31 [0, 5] |
|  | Standard [z-score]: 0.9 ±1.40 [-1.5, +4.1] |
|  | Norm (<1.5): 71 (64.6%)  Subclinical (≥1.5): 15 (13.6%)  Clinical (≥2.0): 24 (21.8%) |
| D  *Depersonalization* | Score [0, 5]: 2.4 ±1.32 [0.0, 5.0] |
|  | Standard [z-score]: 1.8 ±1.47 [-0.8, +6.1] |
|  | Norm (<1.5): 55 (50.0%)  Subclinical (≥1.5): 6 (5.5%)  Clinical (≥2.0): 49 (44.6%) |
| ***BUT, Part B*** |  |
| PST  *Positive Symptom Total* | Score [0, 37]: 22.5 ±9.71 [0, 37] |
| PSDI  *Positive Symptom Distress Index* | Score [0, 5]: 2.8 ±0.85 [0.0, 4.9] |
| *Body part: Mouth* | Score [0, 5]: 1.4 ±0.98 [0.0, 4.2] |
| *Body part: Face Shape* | Score [0, 5]: 1.2 ±1.00 [0.0, 5.0] |
| *Body part: Thighs* | Score [0, 5]: 3.0 ±1.32 [0.0, 5.0] |
| *Body part: Legs* | Score [0, 5]: 2.0 ±1.19 [0.0, 5.0] |
| *Body part: Harms* | Score [0, 5]: 1.8 ±1.19 [0.0, 4.8] |
| *Body part: Moustache* | Score [0, 5]: 1.4 ±1.42 [0.0, 5.0] |
| *Body part: Skin* | Score [0, 5]: 1.6 ±1.32 [0.0, 5.0] |
| *Body part: Blushing* | Score [0, 5]: 1.9 ±1.23 [0.0, 5.0] |
| ***BES*** |  |
|  | Score [0, 46]: 20.5 ±13.33 [0, 45] |
|  | Norm (≤17): 49 (44.1%)  Symptoms (>17): 24 (21.6%)  BED (≥27): 38 (34.2%) |

**BUT,** Body Uneasiness Test; **BES,** Binge Eating Scale; **z-score:** On the basis of Italian standards.

**Supplementary Table 5.** DSM-5 comorbidity.

|  | **N (%)** or **Mean ±SD [min, Max]** |
| --- | --- |
| ***SCID-5-CV*** |  |
| Clinical disorder (any) | Diagnosis: 74 (85.1%) |
|  | Number: 2.2 ±1.51 [0, 6] |
| Clinical anxiety disorder (generalized, panic, social) | Diagnosis: 60 (69.0%) |
|  | Number: 1.1 ±0.89 [0, 3] |
| Diagnosis in classes | Psychotic symptoms (no-diagnosis): 4 (4.6%) |
|  | Bipolar disorder: 3 (3.5%) |
|  | Depressive disorder: 61 (70.1%) |
|  | Generalized anxiety disorder: 38 (43.7%) |
|  | Panic disorder: 35 (40.2%) |
|  | Social anxiety disorder: 18 (20.7%) |
|  | Stress-associated disorder: 10 (11.5%) |
|  | Obsessive-compulsive disorder: 19 (21.8%) |
|  | Substance abuse disorder: 5 (5.8%) |
|  | Attention-associated disorder: 2 (2.3%) |
| ***SCID-5-PD*** |  |
| Personality disorder (any) | Diagnosis: 43 (49.4%) |
|  | Number: 0.8 ±1.04 [0, 4] |
| Personality disorder (cluster A) | Diagnosis: 9 (10.3%) |
|  | Number: 0.1 ±0.31 [0, 1] |
| Personality disorder (cluster B) | Diagnosis: 17 (19.5%) |
|  | Number: 0.2 ±0.47 [0, 2] |
| Personality disorder (cluster C) | Diagnosis: 33 (37.9%) |
|  | Number: 0.5 ±0.71 [0, 3] |
| Paranoid personality disorder | Norm: 75 (86.2%)  Trait: 5 (5.8%)  Disorder: 7 (8.1%) |
| Schizoid personality disorder | Norm: 80 (92.0%)  Trait: 5 (5.8%)  Disorder: 2 (2.3%) |
| Schizotypal personality disorder | Norm: 83 (95.4%)  Trait: 4 (4.6%)  Disorder: 0 (0.0%) |
| Antisocial personality disorder | Norm: 86 (98.9%)  Trait: 0 (0.0%)  Disorder: 1 (1.2%) |
| Borderline personality disorder | Norm: 59 (67.8%)  Trait: 11 (12.6%)  Disorder: 17 (19.5%) |
| Histrionic personality disorder | Norm: 83 (95.4%)  Trait: 3 (3.5%)  Disorder: 1 (1.2%) |
| Narcissistic personality disorder | Norm: 85 (97.7%)  Trait: 2 (2.3%)  Disorder: 0 (0.0%) |
| Avoidant personality disorder | Norm: 50 (57.5%)  Trait: 13 (14.9%)  Disorder: 24 (27.6%) |
| Dependent personality disorder | Norm: 77 (88.5%)  Trait: 4 (4.6%)  Disorder: 6 (6.9%) |
| Obsessive-compulsive personality disorder | Norm: 53 (60.9%)  Trait: 21 (24.1%)  Disorder: 13 (14.9%) |
| Personality disorder NOS | Norm: 86 (98.9%)  Trait: 0 (0.0%)  Disorder: 1 (1.2%) |

**DSM-5,** Diagnostic and Statistical Manual of Mental Disorders, Fifth Edition; **NOS,** Not Otherwise Specified; **SCID-5-CV**, Structured Clinical Interview for DSM-5 Disorders, Clinical Version; **SCID-5-PD,** Structured Clinical Interview for DSM-5 Disorders, Personality Disorders.

**Supplementary Table 6.** Symptom Checklist-90-Revised (SCL-90-R).

| **SCL-90-R scale** | **Mean ±SD [min, Max]** |
| --- | --- |
| GSI  *Global Severity Index* | Score [0, 4]: 1.4 ±0.72 [0.0, 3.6] |
| PST  *Positive Symptom Total* | Score [0, 90]: 54.8 ±20.23 [0.0, 88.0] |
| PSDI  *Positive Symptom Distress Index* | Score [0, 4]: 2.1 ±0.68 [0.0, 3.7] |
| SOM  *Somatization* | Score [0, 4]: 1.3 ±0.91 [0.0, 3.9] |
| O-C  *Obsessive-Compulsive* | Score [0, 4]: 1.6 ±0.91 [0.0, 3.8] |
| I-S  *Interpersonal Sensitivity* | Score [0, 4]: 1.7 ±0.92 [0.0, 3.8] |
| DEP  *Depression* | Score [0, 4]: 2.0 ±0.90 [0.2, 3.8] |
| ANX  *Anxiety* | Score [0, 4]: 1.4 ±0.91 [0.0, 3.7] |
| HOS  *Hostility* | Score [0, 4]: 0.9 ±0.73 [0.0, 3.2] |
| PHOB  *Phobic Anxiety* | Score [0, 4]: 0.8 ±0.83 [0.0, 3.7] |
| PAR  *Paranoid Ideation* | Score [0, 4]: 1.3 ±0.83 [0.0, 3.5] |
| PSY  *Psychoticism* | Score [0, 4]: 0.9 ±0.60 [0.0, 2.9] |

**SCL-90-R,** Symptom Checklist, 90-items, Revised version.

**Supplementary Table 7.** Minnesota Multiphasic Personality Inventory-2 (MMPI-2).

| **MMPI-2 scale** | **Mean ±SD [min, Max]** |
| --- | --- |
| ***Validity scale*** |  |
| L  *Lie* | Score [0, 15]: 5.4 ±2.62 [1, 12] |
|  | Standard [z-score]: +0.1 ±1.02 [-1.6, +2.7] |
| F  *Frequency* | Score [0, 60]: 11.1 ±5.83 [1, 29] |
|  | Standard [z-score]: +1.1 ±1.23 [-1.0, +4.9] |
| K  *Correction* | Score [0, 30]: 12.2 ±4.22 [3, 2] |
|  | Standard [z-score]: -0.5 ±0.89 [-2.5, +1.7] |
| ***Clinical scale*** |  |
| Hs  *Hypochondriasis* | Score [0, 32]: 15.7 ±6.30 [1, 30] |
|  | Standard [z-score]: +1.5 ±1.24 [-1.4, +4.3] |
| D  *Depression* | Score [0, 57]: 33.0 ±6.49 [17, 45] |
|  | Standard [z-score]: +1.8 ±1.13 [-1.0, +3.9] |
| Hy  *Hysteria* | Score [0, 60]: 30.3 ±5.99 [12, 47] |
|  | Standard [z-score]: +1.1 ±1.10 [-2.2, +4.2] |
| Pd  *Psychopathic Deviate* | Score [0, 50]: 26.3 ±5.80 [15, 40] |
|  | Standard [z-score]: +1.5 ±1.07 [-0.6, +4.1] |
| Mf  *Masculinity-Femininity* | Score [0, 56]: 34.8 ±4.88 [2, 45] |
|  | Standard [z-score]: +0.2 ±1.20 [-7.9, +2.7] |
| Pa  *Paranoia* | Score [0, 40]: 15.2 ±4.75 [7, 33] |
|  | Standard [z-score]: +1.4 ±1.26 [-0.8, +6.1] |
| Pt  *Psychasthenia* | Score [0, 48]: 26.8 ±9.30 [4, 43] |
|  | Standard [z-score]: +1.4 ±1.06 [-1.2, +3.2] |
| Sc  *Schizophrenia* | Score [0, 78]: 27.0 ±11.96 [3, 62] |
|  | Standard [z-score]: +1.3 ±1.24 [-1.2, +4.9] |
| Ma  *Hypomania* | Score [0, 46]: 17.4 ±5.54 [6, 32] |
|  | Standard [z-score]: +0.2 ±1.16 [-2.2, +3.3] |
| Si  *Social Introversion* | Score [0, 69]: 38.8 ±10.68 [6, 59] |
|  | Standard [z-score]: +1.1 ±1.19 [-2.6, +3.3] |

**MMPI-2,** Minnesota Multiphasic Personality Inventory, 2nd version; **z-score:** On the basis of Italian standards.
